# Harnessing Bioluminescent Bacteria to Develop an Enzymatic-free Enzyme-linked immunosorbent assay for the Detection of Clinically Relevant Biomarkers

**DOI:** 10.1021/acsami.4c01744

**Published:** 2024-04-23

**Authors:** Liming Hu, Marianna Rossetti, José Francisco Bergua, Claudio Parolo, Ruslan Álvarez-Diduk, Lourdes Rivas, Andrea Idili, Arben Merkoçi

**Affiliations:** † Nanobioelectronics & Biosensors Group, Catalan Institute of Nanoscience and Nanotechnology (ICN2), CSIC and BIST, Campus UAB, Bellaterra 08193, Barcelona, Spain; ‡ Barcelona Institute for Global Health (ISGlobal), Hospital Clínic-Universitat de Barcelona, Barcelona 08036, Spain; § Department of Chemical Sciences and Technologies, University of Rome Tor Vergata, Via della Ricerca Scientifica, Rome 00133, Italy; ∥ Institució Catalana de Recerca i Estudis Avançats (ICREA), Passeig Lluís Companys 23, Barcelona 08010, Spain

**Keywords:** nanoparticles, homogeneous immunoassay, inner filter effect, point-of-care diagnostics, nanomaterials, human IgG, SARS-CoV-2 nucleoprotein

## Abstract

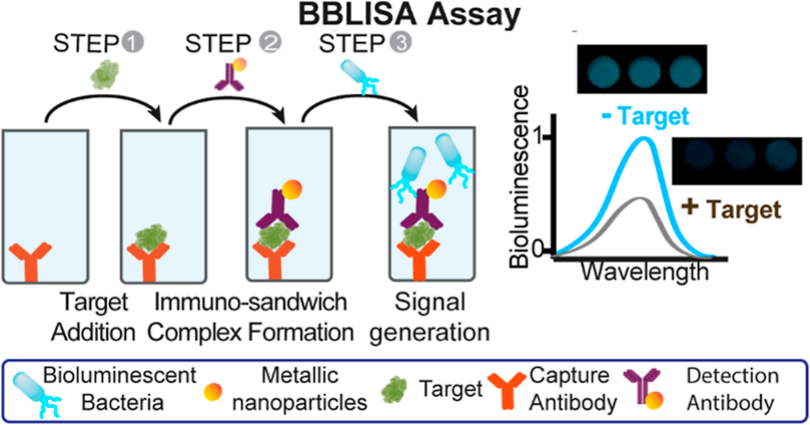

Enzyme-linked immunosorbent assay (ELISA) is the gold standard technique for measuring protein biomarkers due to its high sensitivity, specificity, and throughput. Despite its success, continuous advancements in ELISA and immunoassay formats are crucial to meet evolving global challenges and to address new analytical needs in diverse applications. To expand the capabilities and applications of immunoassays, we introduce a novel ELISA-like assay that we call Bioluminescent-bacteria-linked immunosorbent assay (BBLISA). BBLISA is an enzyme-free assay that utilizes the inner filter effect between the bioluminescent bacteria*Allivibrio fischeri*and metallic nanoparticles (gold nanoparticles and gold iridium oxide nanoflowers) as molecular absorbers. Functionalizing these nanoparticles with antibodies induces their accumulation in wells upon binding to molecular targets, forming the classical immune–sandwich complex. Thanks to their ability to adsorb the light emitted by the bacteria, the nanoparticles can suppress the bioluminescence signal, allowing the rapid quantification of the target. To demonstrate the bioanalytical properties of the novel immunoassay platform, as a proof of principle, we detected two clinically relevant biomarkers (human immunoglobulin G and SARS-CoV-2 nucleoprotein) in human serum, achieving the same sensitivity and precision as the classic ELISA. We believe that BBLISA can be a promising alternative to the standard ELISA techniques, offering potential advancements in biomarker detection and analysis by combining nanomaterials with a low-cost, portable bioluminescent platform.

## Introduction

Since its invention by Engvall and Perlmann in 1971,^1^ the enzyme-linked immunosorbent assay (ELISA) has become one of the most widely used analytical tools in bioanalysis.^2,3^ Its success relies on its ability to detect with high sensitivity almost any biomolecular target (e.g., peptides, proteins, antibodies, hormones, drugs, etc.) directly from biological fluids (e.g., serum, plasma, cell, and tissue extracts, etc.) and within a few hours (between 2 and 8 h).^2,3^ This versatility has made ELISA an indispensable analytical tool for monitoring and evaluating biomarkers,^4,5^ establishing immunoassays platforms as cornerstones in clinical,^6^ pharmaceutical,^7^ food^8^ and environmental analysis.^9^ Despite its success, the recent COVID-19 pandemic has once again highlighted the importance of continued advancements in ELISA and immunoassay formats to meet evolving global challenges and to address new analytical needs across diverse applications.^6,10^ For example, efforts have been made to develop novel enzyme-free signaling mechanisms,^11,12^ or new strategies to increase sensitivity,^13,14^ expand dynamic range,^15^ improve multiplexing capabilities,^16^ and streamline workflows.^17^ The development of new alternative ELISA platforms is still essential to expand the capabilities and applications of immunoassays to enable more accurate and reliable detection of biomolecules.

Over the past four decades, many research groups have attempted to address these technical challenges by proposing novel detection strategies and bioengineering approaches.^6,10^ Regarding the former, most efforts have focused on making the assays more sensitive, accurate, and high throughput by improving one (or more) component of the ELISA assay (e.g., the adsorbent substrate, recognition elements, signaling molecules, and staining reactants). For example, the introduction of nanomaterials and their use as new signaling molecules (e.g., gold nanoparticles (AuNPs), nanorods, nanostars, nanoflowers and silver nanoparticles, etc.) has allowed the classical colorimetric signal to be converted to a fluorescent or chemiluminescent readout (e.g., plasmonic ELISA, bead-based ELISA called Luminex and ELISpot) achieving higher sensitivity and accuracy.^10^ At the same time, the development of new bioengineering approaches has made ELISA easier-to-perform, for example, by reducing the number of steps, the time required to perform the assay, or the volume required for the analysis (e.g., using paper, sliding strips and microfluidic platform).^6^ However, most of the aforementioned studies still rely on the catalytic activity of enzymes,^18^ which requires the purchase of enzyme-modified antibodies and the control of enzyme degradation and loss of activity over time. In contrast, ELISAs that do not rely on enzymatic amplification exhibit several advantages. These include greater stability and longer shelf life, contributing to assay reproducibility and reliability, less expensive, and easier to use (i.e., reduces assay complexity), making the overall platform more robust and potentially more compatible with a wider range of sample types and assay conditions.^11,12,19^

**Figure 1:**
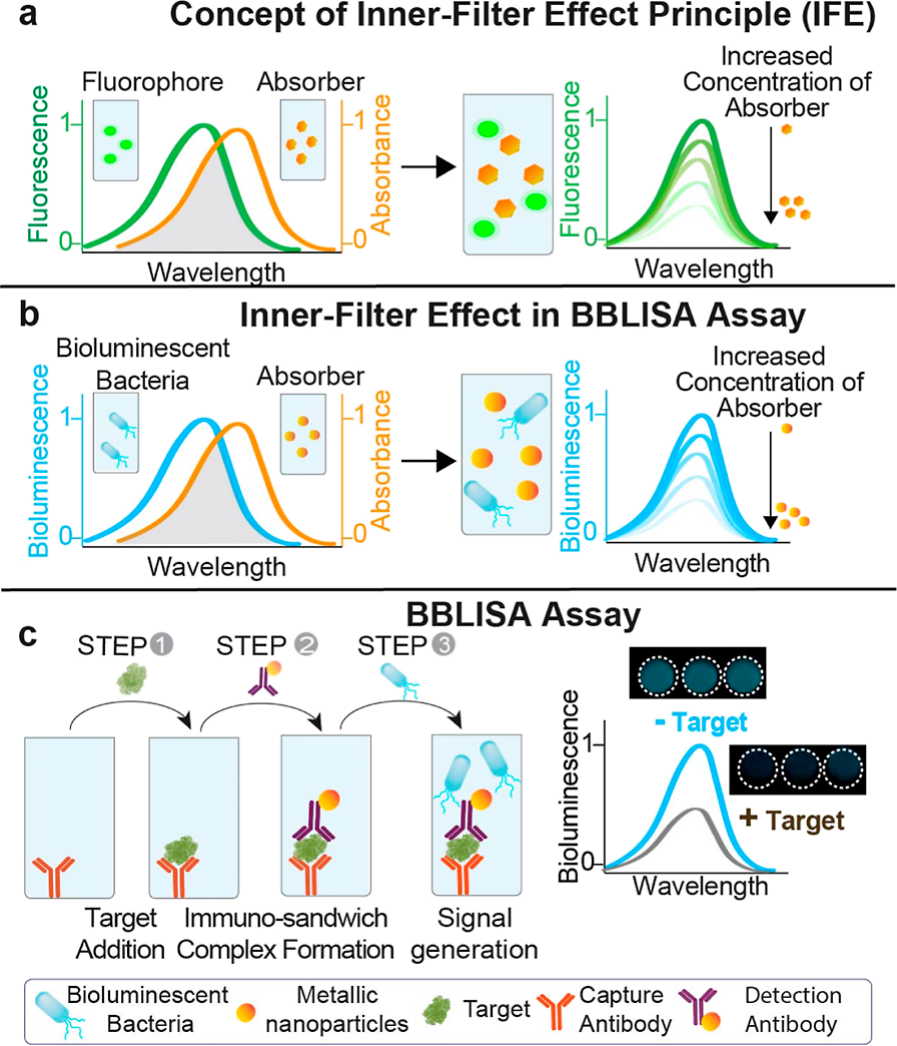
Schematic illustration of the Inner Filter Effect (IFE) and Bioluminescent-Bacteria-Linked Immunosorbent Assay (BBLISA) principles. (a) Inner Filter Effect (IFE). For IFE to occur, the absorption spectrum of the absorber must overlap with the emission spectrum of the fluorophore. As a result, when a fixed concentration of fluorophore is titrated into the well with an increasing concentration of absorber, a decrease in fluorescence emission is observed. (b) IFE in BBLISA assay. In BBLISA, the absorber is a metallic nanoparticle chosen based on its absorption spectrum overlapping the bioluminescence emission spectrum of the bioluminescent bacterium (i.e., *A. fischeri*). Therefore, when a fixed concentration of bioluminescent bacteria is titrated into the well with an increasing concentration of nanoparticles, a decrease in bioluminescence emission is observed. (c) BBLISA assay. The BBLISA assay is based on the classic immunosandwich format, and the protocol consists of the following steps: 1) the target is added to the well and captured by the capture antibody; 2) the detection antibody attached to the surface of a metallic nanoparticle recognizes the target and induces the formation of the immunosandwich complex; 3) the solution containing *A. fischeri* is added to the well and the bioluminescence signal is immediately recorded. Using the immunosandwich format, BBLISA assay generates an optical signal inversely proportional to the concentration of the target.

Motivated by the above arguments, we propose a novel alternative ELISA platform based on the inner filter effect (IFE)^20^ between the bioluminescent bacteria of *Allivibrio fischeri* (*A. fischeri*)^21^ and metallic nanoparticles. We named this new assay: Bioluminescent-bacteria-linked immunosorbent assay (BBLISA). IFE is a radiative energy transfer phenomenon observed in fluorescence measurements that results from the absorption of the excitation and/or emission energy of the fluorophore by the absorber, when the absorption spectrum of the absorber overlaps with the fluorescence excitation or emission spectrum of the fluorophore (Figure 1a).^20^ Of note, such an approach has been reported in previous studies for the development of ELISA platforms, but the generation of the optical signal is still based on the enzymatic activity.^22^ In fact, the enzyme has been exploited to generate a molecular absorber capable of adsorbing the light emitted by a fluorophore. Conversely, in the BBLISA, *A. fischeri* is the species that emits light (i.e., the bioluminescent signal) and the metallic nanoparticles are the species that absorb the emitted light (Figure 1b). More specifically, the presence of the biomolecular target induces the accumulation of the antibody-modified metallic nanoparticles in the well through the formation of the classic immune–sandwich complex (Figure 1c). The subsequent addition of the bioluminescent bacteria to the well allows the generation of an immediate bioluminescent signal whose intensity is inversely related to the number of metallic nanoparticles and thus to the selected target (Figure 1c). Thus, no reactive steps are involved in the generation of the signal. To demonstrate the bioanalytical potential of BBLISA, we successfully employed it for the detection of two biomarkers (the human IgG (HIgG) and the SARS-CoV-2 nucleoprotein (Np)) directly in human serum as a proof of principle. By using different metallic nanoparticles as molecular absorbers, we can modulate the sensitivity of the BBLISA to achieve the same analytical performance as that of a conventional ELISA.

## Results and Discussion

### BBLISA Design and IFE Characterization

The selection of the bioluminescent bacteria responsible for generating the optical signal is a crucial step for the development of the BBLISA platform. While fluorescent molecules can be used for the development of an IFE-based assay,^20^ bioluminescent bacteria offer several practical advantages. First, bacteria do not require external excitation because they generate light through internal biochemical reactions.^21^ This makes them insensitive to photobleaching^23^ and eliminates the need for external excitation sources such as lasers or specific wavelengths of light, making experimental setups cheaper and easier to build. They do not suffer from autofluorescence interference, resulting in better signal-to-noise ratios.^24^ Finally, bacteria are more cost-effective because they do not need to be synthesized or purchased (besides the initial colonies). Indeed, they can be easily made in-house with minimal equipment requirements.^21^ Among the commercially available, naturally bioluminescent bacteria we chose*A. fischeri* because of its advantages: ability to grow at room temperature (20 °C), the reduced risk of contamination due to the high-salt medium used, the availability of inexpensive culture media,^25^ and its stability and activity at room temperature.^26^ In fact, although other bacteria share the same biological mechanism and ability to convert chemical energy into bioluminescence, they (such as *Photorhabdus luminescens* and *Vibrio harveyi*) require higher temperatures (around 30–35 °C) and, therefore, a temperature-controlled experimental setup.^27^ In addition, we have already demonstrated the thermal and storage stability of the *A. fischeri* in previous reports,^21,28^ and as evidence of its bioanalytical properties, which is further supported by the use of *A. fischeri*to develop commercial optical systems (e.g., Microtox) for environmental studies.^29^

**Figure 2:**
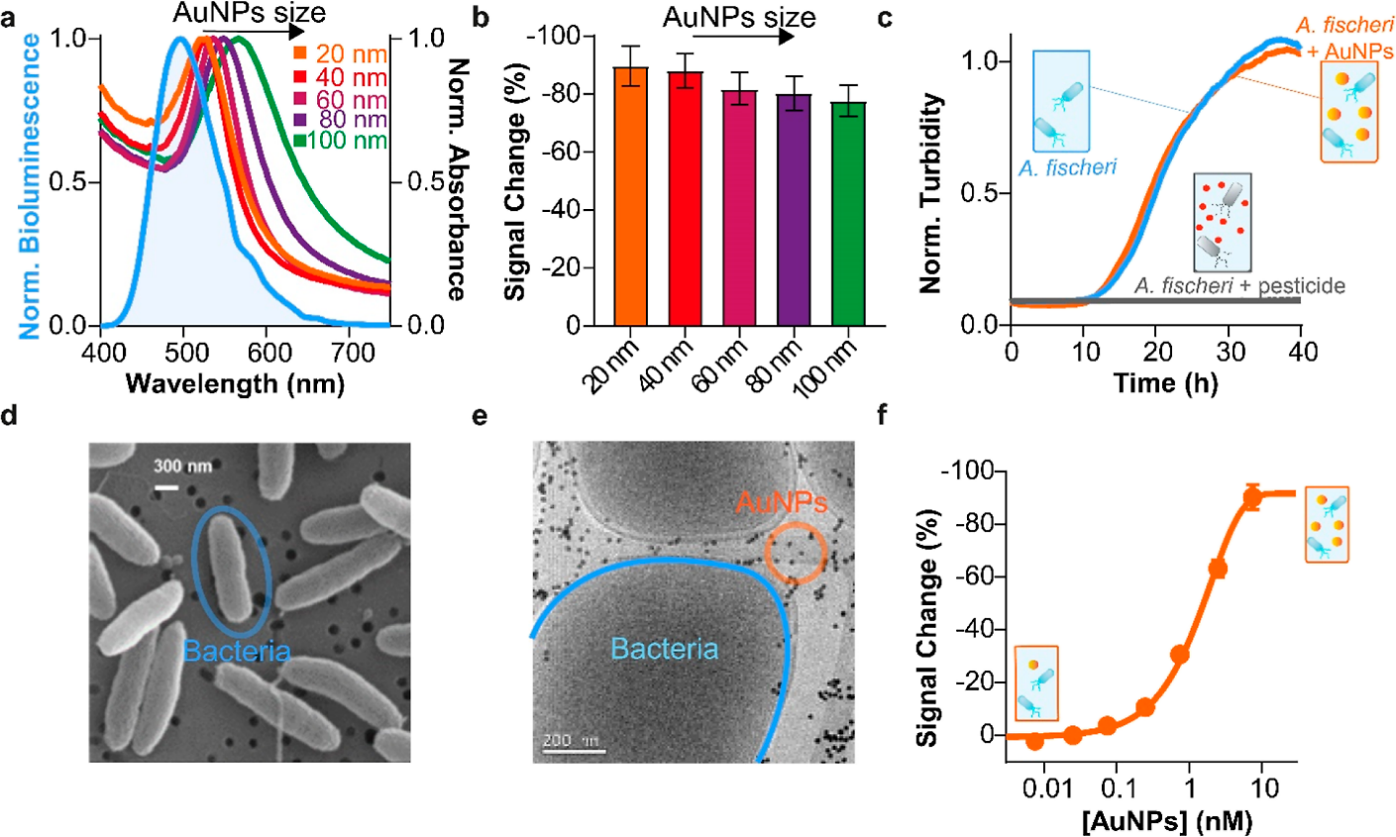
Characterization of the IFE between *A. fischeri* and AuNPs. (a) Normalized emission spectrum of *A. fischeri* (blue) and the absorption spectra of different sizes of AuNPs (20, 40, 60, 80, and 100 nm; from orange to green) are shown. The overlapping capability between *A. fischeri* and AuNPs decreases as the size of AuNPs increases, as indicated by the plasmonic peak red shift associated with larger AuNPs. (b) Bioluminescence signal change of *A. fischeri* as a function of AuNPs size (i.e., 20, 40, 60, 80, and 100 nm). (c) Normalized absorbance at 600 nm of *A. fischeri* in the presence and absence of 20 nm AuNPs (2.5 nM, orange and blue curves) or pesticide tributyltin (100 ng/mL, gray curve) over a time period from 0 to 40 h. (d) SEM image of *A. fischeri*, showing its rod-shaped morphology with terminal flagella. (e) Cryo-TEM image of *A. fischeri* in the presence of AuNPs, indicating that AuNPs are distributed in the culture medium and some even adhere to the wall of *A. fischeri* without affecting their growth. (f) Bioluminescence signal change of *A. fischeri* (1 × 10^9^ CFU/mL) as a function of different concentrations of 20 nm AuNPs. All values reported are the average of three measurements, and error bars reflect standard deviation.

The second step in BBLISA development is the selection of a molecular absorber that can efficiently absorb the light emitted by the bacteria (i.e., *A. fischeri*) (Figure 2a, blue spectrum). This absorber must possess an absorption spectrum overlapping with the emission spectra of *A. fischeri* (Figure 1b). In addition, it must be nontoxic to the bacteria, easy to functionalize with common bioreceptors (e.g., antibodies and aptamers), and must be stable over time. With this in mind, we chose the well-known and widely used AuNPs as a test bed.^30^ More specifically, they exhibit plasmonic (absorption) peaks approximately from 515 to 575 nm (depending on the AuNP diameter)^31^ (Figure 2a). Their synthesis is inexpensive and can be performed using different methods^32^ with small and low-cost laboratory equipment. Finally, they are nontoxic to both bacteria and humans,^33^ and can be easily functionalized with bioreceptors.^34−36^ It is important to highlight that the nontoxicity of AuNPs is crucial because it guarantees that the decrease in bioluminescence signal is due to the IFE and not to the death of bacteria due to the presence of the nanomaterial.

AuNPs can efficiently absorb the light emitted by bacteria, making them an ideal molecular light absorber to demonstrate the signal transduction mechanism of BBLISA. First, we characterized the optical properties of AuNPs to demonstrate their ability to support IFE.^20,37^ We selected a set of AuNPs with different diameters (20, 40, 60, 80, and 100 nm) to take advantage of their diverse absorption spectra (Figure 2a).^38^ We functionalized them with bovine serum albumin (BSA) to prevent their adsorption to the bacteria’s surface and aggregation (see Materials and Methods). This allowed us to create a set of molecular absorbers that share the same chemical composition but have different absorption peaks and, therefore, different overlap with the bioluminescence spectra. Specifically, the AuNPs exhibit their respective absorption peaks at wavelengths of 520, 530, 538, 550, and 568 nm (Figure 2a). We then tested them in the presence of the bioluminescent bacteria to characterize their ability to adsorb the bioluminescent signal. AuNPs induce a progressive and linear decrease of bioluminescence signal as a function of the nanoparticle diameter (Figures 2b and S1), with 20 nm AuNPs showing the highest signal change (90 ± 7%) (relative change in signal upon the addition of the saturating AuNP or target). As expected, this result reflects the ability of AuNPs to overlap the emission spectra of bacteria, but their overlap progressively decreases due to their diameter, which shifts the plasmonic peak to higher wavelengths. Notably, for better comparison, we used concentrations of AuNPs that produced the same absorbance of 0.32 ± 0.01 abs at the corresponding plasmonic peak (Figure S2). This explains why the 20 nm AuNPs perform best as light absorbers because their plasmonic peak better overlaps with the light emitted by *A. fischeri* at 495 nm (Figure 2a, orange spectra), so we selected them for the next development of BBLISA.

We then demonstrate that the decreased bioluminescence signal was solely due to the IFE and not to potential toxicity of AuNPs to the bacteria. We cultured the bacteria in the presence and in the absence of 20 nm AuNPs (concentration of 2.5 nM) and we used the pesticide tributyltin (concentration of 100 ng/mL) as a positive “toxic” control.^21,28,39^*A. fischeri* exhibited the same growth trend with and without AuNPs demonstrating their nontoxicity, as determined by turbidity measurements (using the correlation between absorbance at 600 nm and the number of microorganisms^39^) (Figure 2c, blue and orange curves). On the contrary, the presence of the toxic control, tributyltin, inhibited the bacterial growth due to its toxicity (Figure 2c, gray curve). Similar results were observed for the negative control (only culture media without *A. fischeri*) (Figure S3a, black and red curves). We then used the bioluminescence measurements to monitor the bacterial growth kinetics (Figure S3b). We found again that *A. fischeri* shows the same growth trend for the same bacteria concentration in the presence and absence of AuNPs. However, as expected, the bioluminescent signal was lower in the former due to the IFE produced by the presence of the AuNPs. This result supports previous turbidity measurements. Finally, to further demonstrates the IFE effect, we used SEM to visualize and characterize the bacteria in the absence of nanoparticles (Figure 2d). Then, we used Cryo-TEM images to characterize the interactions between the bacteria and AuNPs (Figure 2e). The Cryo-TEM image indicates that the metal nanoparticles are dispersed in the culture medium and are not absorbed into the bacterial cells. This shows that the decrease in bioluminescence signal is not related to the distance between the bacteria and the AuNPs (as observed for the quenching^40^), but is due to the IFE^40^ that occurs between the AuNPs and the *A. fischeri*.

We next investigated whether AuNPs can suppress the bioluminescence signal in a concentration-dependent manner. Understanding this parameter is crucial to determine if the amount of AuNPs that accumulates on the well surface can induce an IFE strong enough to produce a detectable signal change. To better understand this parameter from a quantitative perspective, we estimated the number of antibodies adsorbed on the well surface. To do this, we use the packing density value ((1.50 ± 0.06) × 10^12^ molecules per cm^–2^) reported in a recent study characterizing the physiobsorbed antibody layer on gold surfaces.^41^ Using this value, we calculated the antibodies adsorbed on the well surface (0.32 cm^2^, flat bottom), which corresponds to 4.8 × 10^11^ molecules. Assuming a binding ratio of 1:1 (one nanoparticle per antibody) we converted the previous value to molar units. Using the volume of the solution (100 μL), we estimated the concentration in molarity (mol/L), and this value corresponds to approximately 8 nM. We then measured the bioluminescence emitted by the bacteria (1 × 10^9^ cfu/mL) in the presence of increasing concentrations of 20 nm AuNPs (from 0.0075 to 7.5 nM) (Figure 2f). The optical data indicate that concentrations of AuNPs below 0.075 nM do not cause a relevant decrease in the bioluminescence signal (compared with the bioluminescence intensity in the absence of AuNPs). Conversely, AuNPs concentrations higher than 0.25 nM cause a progressive decrease in the intensity of the bioluminescence signal. Specifically, 0.25 nM of AuNPs leads to an 11% decrease in bioluminescence intensity, while 7.5 nM concentration allows to reach 90% of bioluminescence suppression (Figure 2f). Therefore, the AuNPs can effectively achieve an IFE in the nanomolar range and can be adapted to support the BBLISA platform.

Finally, we optimized the concentration of bacteria to achieve the highest bioluminescence signal change. We tested four different concentrations of bacteria (5 × 10^7^, 1 × 10^8^, 5 × 10^8^ and 1 × 10^9^ cfu/mL) in the presence of three concentrations of AuNPs (0.25, 0.75, and 2.5 nM) (Figure S4a). As expected, the addition of AuNPs induces a decrease in the bioluminescence signal in all suspensions, with the highest raw signal change at the highest bacterial concentration (1 × 10^9^ cfu/mL). To ensure that the analytical signal was independent to changes in bacterial concentration, we converted the raw bioluminescence signal to a signal change (%) (see Experimental Methods for more details). This gives us the same signal change value of ∼25, ∼50 and ∼73% in the presence of 0.25 0.75, and 2.5 nM AuNPs, respectively, independently of the bacterial concentration used (Figure S4b). This simple conversion of the raw signal change allows to make the total signal change (%) to be independent of the initial bacterial concentration, as it is only related to the amount of AuNPs present in the solution. This is critical for the development of a diagnostic device because the final signal change can be directly related to the target concentration even if the illumination (in this case the bioluminescence generated by the bacteria) varies from test to test.

### BBLISA Based on AuNPs

**Figure 3:**
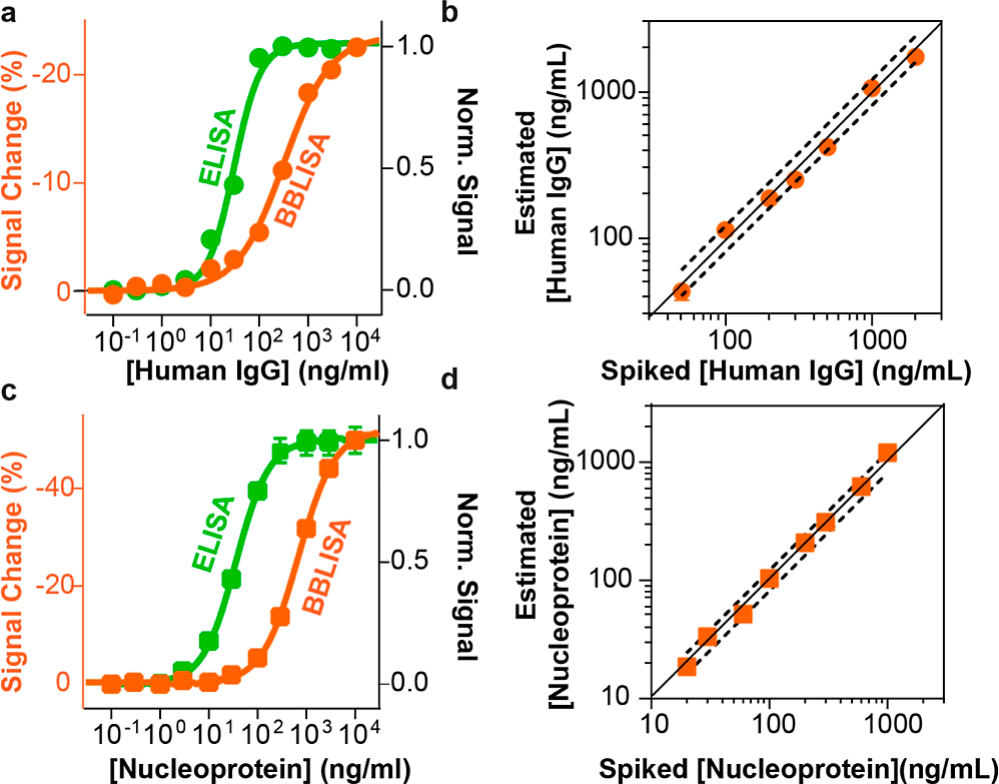
Detection of human IgG and nucleoprotein of SARS-CoV-2 in human serum based on ELISA and BBLISA_AuNPs. (a) Calibration curves for the detection of HIgG (from 0.1 to 3000 ng/mL) based on BBLISA_AuNPs (orange) and ELISA (green curve). (b) Accuracy of BBLISA_AuNPs within ±20% (black dashed line) for the detection of HIgG from serum samples in the range of 50–2000 ng/mL. (c) Calibration curves for the detection of SARS-CoV-2 nucleoprotein (from 0.1 to 3000 ng/mL) based on BBLISA_AuNPs (orange curve) and ELISA (green curve). (d) Accuracy of BBLISA_AuNPs within ±20% (black dashed line) for the detection of SARS-CoV-2 nucleoprotein from serum samples in the range of 20–1000 ng/mL. Error bars reported for BBLISA and ELISA measurements reflect standard deviations derived from three independent wells.

BBLISA represents an alternative bioanalytical platform because it can detect the presence of the selected target in its clinically relevant range. After demonstrating the IFE between AuNPs and *A. fischeri* in solution, we decided to use it for the development of the BBLISA platform. To synthesize AuNPs we used a protocol developed and optimized in our recently published studies,^42,43^ based on the Turkevich method.^44^ We characterized the AuNPs using TEM images^42,43^ (Figure S5a). As expected, the AuNPs exhibited a spherical morphology, homogeneous size, an overall average diameter corresponding to 19.6 ± 0.9 nm, and a monodisperse size distribution (Figure S5a,b, Table S1). We then functionalized them with a primary antibody (i.e., antihuman IgG antibody) capable of detecting human immunoglobulin antibody (IgG), a common serologic biomarker associated with infection and inflammation.^45^ Of note, we selected the functionalization process and antibodies that have been characterized in our previous studies and successfully adapted to support bioanalytical platforms (i.e., LFAs^43,46,47^ and electrochemical immunoanalysis^48^). We used dynamic light scattering (DLS) and Z-potential to confirm that the AuNPs were functionalized and fully covered with the anti-HIgG after the conjugation process (Figure S5c and Table S1), as previously reported.^47^ Next, we characterized the analytical performance of the BBLISA (Figure 3) by collecting the bioluminescent signal in the presence of increasing concentrations of the target (Figure 3a, orange curve; and Figure S6a). As expected, the presence of IgG induces the formation of the immune–sandwich complex, which decreases the bioluminescent signal (up to −23.2 ± 0.3%) resulting in the expected sigmoidal calibration curve, as generally observed in immunoassay platforms.^46,49,50^ Fitting the data with a four-parameter logistic equation we estimated an inflection point (IC_50_) of 320 ± 40 ng/mL, a dynamic range of 25 to 2500 ng/mL (i.e., the concentration range that induces a signal change of 10 to 90%^46^), and a limit of detection (LOD) of 2.0 ± 0.4 ng/mL (Figures 3a, S6a and Table 1). These analytical parameters allow to easily measure the broad clinically relevant IgG range (from 7.0 to 16.0 mg/mL).^51^

**Table 1:** Analytical Performance of the ELISA, BBLISA_AuNPs, and BBLISA_Au–IrO_2_ NFs for the Detection of HIgG and SARS-CoV-2 Nucleoprotein in Human Serum

analyte	parameters	ELISA (ng/mL)	BBLISA_AuNPs (ng/mL)	BBLISA_Au–IrO_2_ NFs (ng/mL)
human IgG	LOD	0.6 ± 0.1	2.0 ± 0.4	0.4 ± 0.1
	LOQ	1.3 ± 0.1	32 ± 5	1.7 ± 0.2
	IC_50_	33 ± 4	320 ± 40	24 ± 5
	linear range	8.0–100	25–2500	2–250
nucleo-protein	LOD	0.6 ± 0.1	25 ± 6	0.6 ± 0.2
	LOQ	1.4 ± 0.1	66 ± 6	0.8 ± 0.1
	IC_50_	37 ± 1	690 ± 20	28 ± 2
	linear range	6–190	100–3700	3–255

BBLISA demonstrates clinically relevant accuracy when challenged with a real biological fluid. To evaluate the accuracy of the method mimicking a clinical scenario, we used serum samples spiked with known concentrations of the IgG target. Specifically, we spiked seven different concentrations of HIgG into HIgG-depleted human serum (i.e., human serum that has been treated to remove naturally occurring IgG antibodies). Using the previously obtained calibration curve (Figure 3a), we precisely estimated the concentration of spiked HIgG with a relevant error of ±20% from 30 to 3000 ng/mL. Indeed, seven of spiked concentrations are perfectly positioned on the diagonal line of the graph indicating an excellent correlation between spiked and estimated concentrations (Figure 3b). These data are further supported by the spiked recoveries ([estimated analyte]/[spiked analyte] × 100%), whose values are between 80 and 120% (Table S2) and were calculated using the previously estimated concentrations (Figure 3b). Therefore, BBLISA can accurately quantify the presence of the target even when tested in a real biological fluid such as human serum.

To better characterize the analytical performance of the BBLISA, we compared it with a classical colorimetric ELISA that relies on horseradish peroxidase (HRP) to generate the optical signal. To obtain a direct comparison between the two immunoassays, we used the same bioreceptors (i.e., immune-sandwich), reagents, and incubation times involved in the various steps of functionalization of the 96-well plates (e.g., coating, target incubation, washing; Figure 1c). We found that the ELISA has a higher sensitivity than the BBLISA (Figure 3a, green curve). For example, the IC_50_ is 1 order of magnitude lower (33 ± 4 ng/mL) and the LOD is 0.6 ± 0.1 ng/mL (Figures 3a and S7a and Table 1). We believe that the observed lower analytical performance of BBLISA is due to the reduced ability of the IFE to generate a change in the optical signal with respect to enzymatic amplification (Table 1).

To demonstrate the generalizability of our bioluminescent platform, we used BBLISA to detect a second different clinical target: the nucleoprotein of SARS-CoV-2.^52^ Again, we selected a pair of antibodies that we have recently characterized that are able to specifically recognize the target by forming an immune–sandwich complex.^42^ The BBLISA can efficiently detect the nucleoprotein with a higher signal change (up to −50.2 ± 0.1%), and we estimated an IC_50_ and LOD of 690 ± 20 and 25 ± 6 ng/mL, respectively (Figure 3c, orange curve; Figure S6b and Table 1). Our assay can precisely estimate nucleoprotein concentrations with good precision and accuracy (±20%) in its clinical range.^52^ To demonstrate this, we challenged BBLISA with human serum samples spiked with eight different concentrations of nucleoprotein, and we estimated the target concentration (Figure 3d) using the previous calibration curve (Figure 3c). We then calculated the spike recovery using the same approach as that for the previous target (Table S3). Finally, we compared the analytical performance of the BBLISA with that of the ELISA (Figure 3c, green curve). As observed for IgG, the ELISA displays higher sensitivity, and we estimated an IC_50_ and LOD of 37 ± 1 and 0.6 ± 0.1 ng/mL, respectively (Figure S7b and Table 1).

### BBLISA Based on Au–IrO_2_ NFs

**Figure 4:**
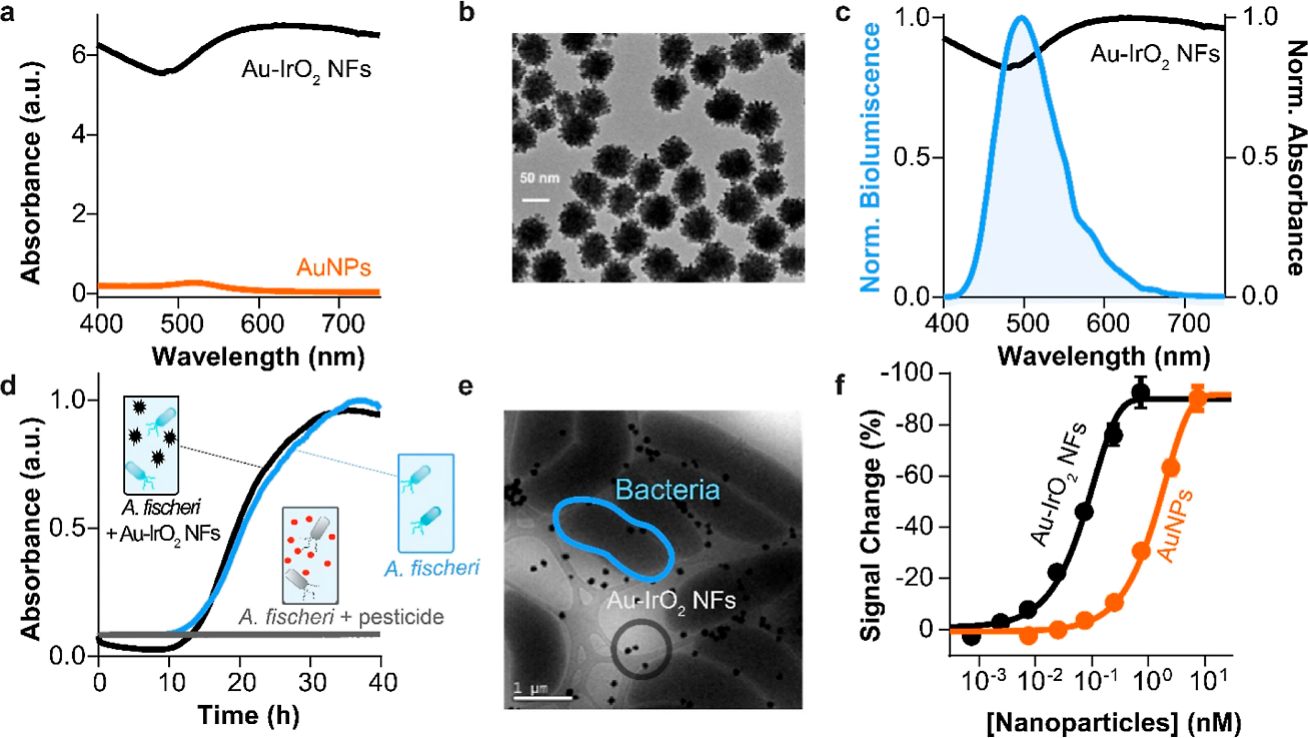
Optical characterization of IFE between *A. fischeri* and Au–IrO_2_ NFs. (a) Absorbance spectrum of AuNPs (orange) and Au–IrO_2_ NFs (black) performed at the same concentration (1.25 nM). (b) TEM of Au–IrO_2_ NFs displaying overall spherical morphology and a surface presenting a highly tortuous branched structure. (c) Normalized bioluminescence emission spectrum of *A. fischeri* (10^9^ cfu/mL, blue line) and absorbance spectrum of Au–IrO_2_ NFs (black line). (d) Normalized turbidity signal (absorbance at 600 nm) of *A. fischeri* in the presence and absence of Au–IrO_2_ NFs (0.24 nM, black and blue curves) or pesticide tributyltin (100 ng/mL, gray curve) from 0 to 40 h. (e) Cryo TEM image of *A. fischeri* with Au–IrO_2_ NFs. (f) Bioluminescence signal change of *A. fischeri* (1 × 10^9^ cfu/mL) as function of different concentrations of nanoparticles (i.e., AuNPs in orange, Au–IrO_2_ NFs in black). All values reported are the average of three measurements, and error bars reflect standard deviations.

To improve the analytical performance of BBLISA, we have investigated new molecular absorbers with superior absorption properties and a spectrum that better overlaps the bioluminescence spectra of *A. fischeri*. The goal is to improve the IFE effect to increase the sensitivity of the BBLISA. For this purpose, we decided to use gold–iridium oxide nanoflowers (Au–IrO_2_ NFs) for two reasons: they have a higher extinction coefficient and surface area than those of AuNPs,^53^ and we have recently optimized their synthesis and functionalization and fully characterized their morphology, composition, size, and stability.^47^ In addition, we demonstrate their ability to support target biorecognition in a paper-based sensing platform.^47^ Therefore, based on our previous experience, we synthesized Au–IrO_2_ NFs, and we characterized them using TEM images (Figure 4b). As expected, the nanoflowers exhibit a spherical morphology with a highly tortuous branched structure (Figure 4b) and an overall average diameter of 62.3 ± 5.3 nm resulting in a monodisperse size distribution (Figure S8a).^47^ Next, we demonstrate their superior absorption properties by comparing the UV–vis spectra of Au–IrO_2_ NFs and AuNPs performed at the same nanoparticle concentrations (Figure 4a). Nanoflowers exhibit 25 times higher molar extinction coefficient than 20 nm of AuNPs, which is due to their surface flower-like branching morphology and their hybrid composition of gold and iridium.^53^ Additionally, their spectrum overlaps better with the emission spectra of bacteria, ensuring higher absorption of bioluminescence (Figure 4c). The collected data are in perfect agreement with previous studies.^47,53^

Au–IrO_2_ NFs have no toxic effect on bacteria and can enhance the IFE. We used turbidity measurements to investigate the toxicity of these nanoparticles by monitoring the growth of *A. fischeri* in the presence and in the absence of Au–IrO_2_ NFs. Similar to what was observed for AuNPs (Figure 2c), the bimetallic nanoparticles exhibit the same behavior, affecting only the bioluminescent signal (Figures 4d and S9). Cryo-TEM images of *A. fischeri* in the presence of Au–IrO_2_ NFs clearly showed that the nanoparticles are distributed around the bacteria or in the culture medium and are not absorbed into the bacterial cells (Figure 4e). This observation further supports our proposed mechanism, which is based on the IFE and is not dependent on the distance between the light source and the filter particles.^40^ Finally, we characterized the IFE by measuring the bioluminescent signal produced by the bacteria (1 × 10^9^ cfu/mL of *A. fischeri*) in the presence of increasing concentrations of Au–IrO_2_ NFs (Figure 4f, black curve). The optical data demonstrate that NFs can suppress the bioluminescence signal more efficiently, requiring lower concentrations compared to AuNPs. For example, a 6-fold concentration of initially synthesized Au–IrO_2_ NFs (0.73 nM) induces a signal change of −92.7% which would require a concentration of AuNPs at least ten times higher to obtain. Like the previous system, the signal change is reproducible and is not affected by the relative concentration of *A. fischeri* (Figure S10).

**Figure 5:**
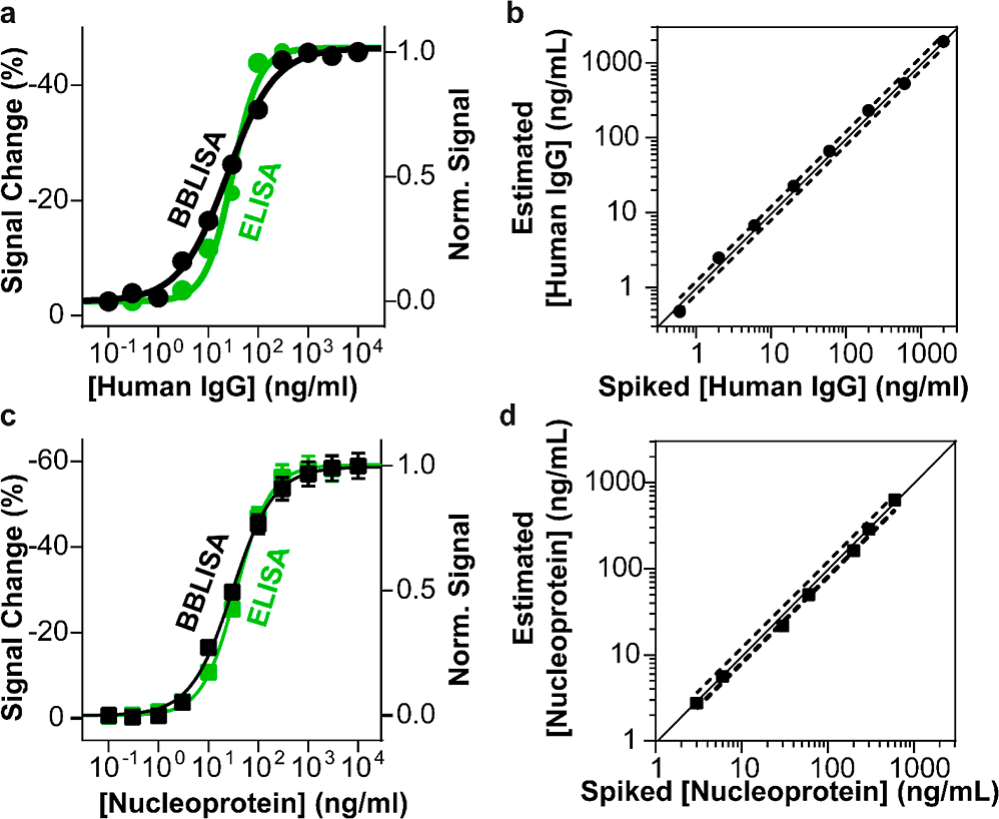
Detection of HIgG and nucleoprotein of SARS-CoV-2 in human serum based on ELISA and BBLISA_Au–IrO_2_ NFs platform. (a) Calibration curves for the detection of HIgG (from 0.1 to 3000 ng/mL) based on ELISA (green curve) and BBLISA_Au–IrO_2_ NFs (black curve). (b) Accuracy of BBLISA_Au–IrO_2_ NFs within ±20% (black dashed line) for the detection of HIgG from serum samples in the range of 0.6–2000 ng/mL. (c) Calibration curves for the detection of SARS-CoV-2 nucleoprotein (from 0.1 to 3000 ng/mL) based on ELISA (green curve) and BBLISA_AuNPs (black curve). (d) Accuracy of BBLISA_Au–IrO_2_ NFs within ±20% (black dashed line) for the detection of SARS-CoV-2 nucleoprotein from serum samples in the range of 3–600 ng/mL. Error bars reported for BBLISA and ELISA measurements reflect standard deviations derived from three independent wells.

The high light absorption capacity of Au–IrO_2_ NFs and their ability to induce a stronger IFE can be utilized to improve the BBLISA platform. We performed BBLISA experiments for the detection of HIgG and SARS-CoV-2 nucleoproteins (Figure 5). First, we used DLS and Z-potential to demonstrate that the Au–IrO_2_ NFs are functionalized and coated with the corresponding primary antibody after the conjugation process (Figure S8b and Table S1). We then evaluated the analytical performance of the Au–IrO_2_ NFs-based BBLISA against increasing concentration of IgG (Figures 5a and S11a). By fitting the curve of signal change versus IgG concentration, we estimated IC_50_ and LOD values of 24 ± 5 and 0.4 ± 0.1 ng/mL, respectively, which are lower than those obtained with the AuNPs- based BBLISA (LOD = 2.0 ± 0.4 ng/mL; Table 1). This indicates that we have significantly improved the sensitivity of our platform by achieving a 5-fold lower detection limit. In addition, we used BBLISA to accurately estimate spiked HIgG concentrations in HIgG-depleted human serum using the same approach as the previous BBLISA based on AuNPs (Figure 5b and Table S2). The recoveries of different spiked concentrations are between 80% and 120%, and the relative standard deviations are always less than 20% (Table S2). The improvement of the analytical performance can be further demonstrated by comparing the Au–IrO_2_ NFs-based BBLISA with the classical ELISA (Figure 5a, green curve), where they show lower IC_50_ and LOD values than ELISA (Figure 5a, and Table 1). Finally, we performed a BBLISA to detect the nucleoprotein of SARS-CoV-2. As observed for the previous target, the Au–IrO_2_ NFs-based BBLISA could detect the SARS-CoV-2 nucleoprotein with a lower LOD (0.6 ± 0.2 ng/mL) and IC_50_ (28 ± 2 ng/mL) (Figures 5c and S11b) with respect to the AuNPs-based BBLISA. The recoveries of seven different spiked concentrations are between 80% and 120% and the relative standard deviations are always below 20% (Figure 5d and Table S3). The overall analytical performance results are again comparable even better than those obtained with the standard ELISA (IC_50_ = 37 ± 1 ng/mL, LOD = 0.6 ± 0.1 ng/mL and dynamic range from 6 to 190 ng/mL, ∼32-fold), and also show a wider dynamic range (from 3 to 255 ng/mL, ∼85-fold) (Figures 5c, S11b and Table 1).

## Conclusions

In summary, we have developed a novel bioluminescence, enzyme-free immunoassay based on the IFE between bioluminescent bacteria and metallic nanoparticles. We have named this newly developed assay BBLISA. By using AuNPs as molecular absorbers, we were able to demonstrate the signal transduction mechanism of BBLISA. We selected 20 nm AuNPs due to their ability to generate the highest signal change. To demonstrate the clinical potential of this platform, we successfully applied BBLISA to detect HIgG and SARS-CoV-2 nucleoprotein in human serum samples as a proof of principle. We demonstrate the sensitive and selective detection of the selected biomarkers within their clinically relevant range. We compared our platform with a classical colorimetric ELISA, and we observed a lower sensitivity of the BBLISA. To improve the analytical performance, we exploited the inherent modularity and versatility of the BBLISA, which make the platform easily adaptable for the use of novel molecular absorbers with superior optical properties. We used bimetallic nanoparticles Au–IrO_2_ NFs as new molecular absorbers to enhance the sensitivity of BBLISA. As AuNPs, they are nontoxic to the bacteria, they are stable, and easy to functionalize with bioreceptors, but unlike them, Au–IrO_2_ NFs showed higher absorption properties. Using these nanoparticles, we achieved a higher sensitivity for the detection of the selected biomarkers, demonstrating the clinical potential of BBLISA as an alternative to the current ELISA.

The reported data demonstrate the ability of our proposed platform to detect clinically relevant biomarkers with the same sensitivity as that of conventional ELISA. BBLISA represents a valid bioanalytical alternative because, in addition to high analytical performance, it is versatile and modular, offering other advantages (Tables S4 and S5). For example, it is less expensive because it does not require enzyme-labeled antibody/streptavidin and/or chromogenic substrates for the signal generation. Because the bacteria can emit light in high yields, the bioluminescence signal can be easily collected using a commercially available smartphone, allowing the device to support low-cost, optical devices.^54^ In addition, the BBLISA platform is also faster because it requires fewer steps; for example, because the signal generation is not based on an enzymatic reaction, we do not need enzyme (i.e., HRP) secondary antibody incubation nor to stop the reaction at a specific time. In addition, the growth of the bacteria in microbiological culture media allows for continuous in-house regeneration of the “optical substrate” without the need for expensive equipment. In our previous study,^21^ we described how to use agitation and temperature to standardize the bacterial growth. More importantly, we show that their production can be tracked using low-cost, smartphone-based optical devices to collect absorbance at 600 nm and bioluminescence signal.^21,54^

In addition to these advantages, the novel enzyme-free transduction mechanism may allow the use and integration of novel nanomaterials other than metallic nanoparticles used as a proof of principle. For example, nanomaterials with a higher absorbance could further increase the magnitude of the IFE and the relative signal change. This could push the detection limit below that of the classical ELISA assay. The ability to select nanomaterials with different optical properties will allow the dynamic range of the assay to be programmed as a function of the clinically relevant range of the selected target. In addition, fluorescent nanomaterials could be used to convert the assay from a signal-off to a signal-on platform. In this scenario, the light emitted by the bacteria will excite the surface-attached nanomaterial, triggering its fluorescence. If achieved, this could lead to a synergistic integration of nanomaterials with living organisms to improve our understanding of their biological properties and develop improved optical bioanalytical platforms.

## Experimental Methods

### Chemicals and Reagents

Tetrachloroauric acid trihydrate (HAuCl_4_·3H_2_O 99.9%), iridium(III) chloride hydrate (IrCl_3_·*x*H_2_O 99.9%), trisodium citrate dihydrate, phosphate buffered saline (PBS) tablets, disodium hydrogen phosphate heptahydrate, monosodium phosphate, sodium bicarbonate, sodium carbonate anhydrous, boric acid, sodium tetraborate decahydrate, sodium chloride, hydrochloric acid, sodium hydroxide, BSA, Tween-20, 3,3,5,5-tetramethylbenzidine (TMB, T0440 & T4444), sulfuric acid, HIgG from human serum (I2511), antihuman IgG (produced in goat; I1886), and biotinylated antihuman IgG (γ-chain specific) (produced in goat; B1140) were purchased from Sigma-Aldrich (St. Luis, MO, USA). Human immunoglobulin depleted serum was purchased from Celprogen (Torrance, CA, USA). SARS-CoV-2 nucleocapsid-his recombinant protein (40588-V08B), monoclonal mouse antinucleoprotein antibody (40143-MM08) and polyclonal rabbit antinucleoprotein antibody (40588-T30) was supplied by Sino Biological. Normal human serum (S1-100 ML) was purchased from Sigma-Aldrich (St. Luis, MO, USA). Tributyltin, tryptone, yeast extract, glycerol for molecular biology, agar, sucrose and casein hydrolysate were purchased from Sigma-Aldrich (St. Luis, MO, USA).

### Preparation of the Bacterial Culture Medium and Buffers

Marine broth (MB) medium was prepared by dissolving 5 g of tryptone, 20 g of sodium chloride, 3 g of yeast extract, and 3 mL of glycerol in 1000 mL of Milli-Q H_2_O and then autoclaved for 30 min at 121 °C. One PBS tablet (Sigma, P4417-100TAB) was dissolved into 200 mL of Milli-Q H_2_O to get 0.01 M, pH 7.4 of PBS buffer. The washing buffer of 0.05% PBST was prepared by adding 0.5 mL Tween-20 to 999.5 mL of 0.01 M, pH 7.4 of PBS buffer. The 0.05 M, pH 9.6 carbonate-bicarbonate buffer (CBS) was prepared by dissolving 2.88 g of sodium bicarbonate and 1.666 g of sodium carbonate (anhydrous) to 800 mL of Milli-Q H_2_O and then using Milli-Q H_2_O adjusting total volume to 1000 mL.

### Storage and Production of Bioluminescent Bacteria

*A. fischeri* (ATCC 700601) was purchased from the ATCC collection (Manassas, VA, USA) and stored at −80 °C. *A. fischeri* was cultured according to the protocol previously established by our group.^21^ Initially, 25 μL of a stock of *A. fischeri* was taken from −80 °C storage and thawed at room temperature for a minimum of 10 min. This 2.5 μL aliquot was then added to 25 mL of MB medium in an Erlenmeyer flask, and the solution was cultured at room temperature for 18–24 h with continuous orbital shaking at 135 rpm using a SSM1 Stuart mini-orbital shaker (Staffordshire, United Kingdom). If the culture needed to be renewed, 2.5 μL of a 24 h-old bacterial culture was added to 25 mL of MB medium, and the process was repeated. The concentrations of bacterial suspensions were estimated by analyzing the optical density value at 600 nm (OD_600_) using the Fisherbrand Cell Density Meter and the software provided by Agilent Genomics (https://www.chem.agilent.com/store/biocalculators/calcODBacterial.jsp). For storage, bacteria are rapidly frozen at concentrations of 10^8^ cfu/mL at −80 °C (measured by OD600). While viable bacteria can be maintained at −20 °C, long-term stability is ensured at −80 °C. The recovery of bioluminescence is rapid after thawing the bacteria at 25 °C for 30 min in a small amount of MB medium. To reproduce bacterial cultures, 2.5 μL of recently thawed bacteria can be inoculated into 25 mL of suitable growth media, allowing them to grow for 20–24 h until a concentration of 10^9^ cfu/mL is reached.

### Synthesis of Metallic Nanoparticles

To characterize the BBLISA sensing mechanism (Figure 2), we used commercial AuNPs of different sizes (20, 40, 60, 80, and 100 nm) that were purchased from nanoComposix (Czech Republic) and stored away from light at 4–6 °C in 2 mM sodium citrate solution. For the BBLISA experiments (Figure 3), we synthesized AuNPs of 20 nm using a revised Turkevich synthesis method.^44^ Specifically, we added 8 mL of a 1% HAuCl_4_ solution (25 mM) to an Aqua Regia-clean Erlenmeyer flask and adjusted the volume to 400 mL using milli-Q water. The solution was heated to boiling point, and 10 mL of 1% (w/v) trisodium citrate dihydrate was added with vigorous stirring using an IKA Magnetic Stirrers (Spain). The solution was kept at the same condition for 10 min until the color changed from light yellow (HAuCl_4_ color) to deep blue and eventually to wine red. The solution was left to cool down to room temperature with only light stirring (100 rpm), and then stored at stored away from light at 4–6 °C in 0.85 mM sodium citrate solution.

For the BBLISA experiments (Figures 4 and 5), gold iridium oxide nanoflowers (Au–IrO_2_ NFs) was synthesized following a synthesis method we recently developed.^47^ Initially, we heated 25 mL of a solution of sodium citrate (2.5 mM) until it reached a boiling point. Next, we mixed 1770 μL of HAuCl_4_·3H_2_O (12 mM) with 442.5 μL of IrCl_3_·*x*H_2_O (12 mM) and adjusted the solution to 5 mL using milli-Q water. This solution was added in a single step to a boiling sodium citrate solution. Boiling was continued for an additional 2 min, during which time the solution’s color changed from pale green to petrol blue. We then cooled the suspension to room temperature under continuous stirring and stored it at 4 °C until further use.

### Optical Characterization of *A. fischeri* and Metallic Nanoparticles

The multimode microplate readers SpectraMax iD3 from Molecular Devices (San José, CA, USA) was used to collect the bioluminescence and absorption spectra of bacteria and metallic nanoparticles, and to collect the colorimetric and bioluminescence signal during ELISA and BBLISA experiments, respectively. Microplates were purchased from Thermo Fisher (Spain), including transparent 96-well microplates (10078850), transparent and white immuno nonsterile 96-well microplates (10777621 and 10396181), and white sterilized cell culture 96-well microplates (10072151). To collect the bioluminescence spectra of *A. fischeri* bacteria (Figure 2a), 100 μL of a bacterial suspension with a concentration of 1 × 10^9^ cfu/mL was added to white 96-well microplates. Bioluminescence was detected every 2 nm from 400 to 700 nm. To collect the absorption spectra of metallic nanoparticles (Figures 2a and 4a,b), 100 μL of different sizes of AuNPs (20, 40, 60, 80, and 100 nm) and Au–IrO_2_ NFs were pipetted into transparent 96-well microplates. Absorbance was detected every 2 nm from 400 to 700 nm.

### Growth of *A. fischeri* in the Presence of Metallic Nanoparticles

To characterize the IFE between *A. fischeri* and metallic nanoparticles (Figures 2 and 4), the naked nanoparticles were first coated with BSA. Specifically, 1 mL of synthesized nanoparticles was mixed with 100 μL of 10% (w/v) BSA in H_2_O and incubated for 30 min at room temperature under shaking at 550 rpm. The conjugates were then centrifuged at room temperature using different speeds depending on the sizes of the AuNPs and Au–IrO_2_ NFs. More specifically, 1000 μL of 20 nm AuNPs were centrifuged at 14,000 rpm (17 530 rcf) for 20 min, 40 nm AuNPs at 10 000 rpm (8944 rcf) for 20 min, 60 nm AuNPs at 8000 rpm (5724 rcf) for 15 min, 80 nm AuNPs at 5000 rpm (2236 rcf) for 10 min, 100 nm AuNPs at 3500 rpm (1096 rcf) for 10 min, and Au–IrO_2_ NFs at 6000 rpm (3220 rcf) for 10 min. The supernatant was removed, and the pellets were resuspended by using 500 μL of MB media. For the bioluminescence measurements, 50 μL of BSA coated nanoparticles were mixed with 50 μL of *A. fischeri* (10^9^ cfu/mL) in 96-well microplates.

To obtain bioluminescence titration curves of *A. fischeri* (1 × 10^9^ cfu/mL) (Figures 2d and 4d), metallic nanoparticles were conjugated with BSA using the previous procedure. Specifically, 20 nm AuNPs conjugates were prepared at different concentrations (7.5 25, 75, 0.25, 0.75, 2.5, and 7.5 nM) in MB. Additionally, different concentrations of Au–IrO_2_ NFs conjugates (0.73, 2.4, 7.3 pM. Twenty-four pM, 73 pM, 0.24 nM and 0.73 nM) were prepared in MB. And then 50 μL of AuNPs or Au–IrO_2_ NFs conjugates at each concentration were mixed with 50 μL of *A. fischeri* (10^9^ cfu/mL) in the 96-well microplates. Bioluminescence signals were collected from three different wells for each metallic nanoparticles’ conjugates concentration.

### Growth Curves of *A. fischeri* in the Presence of AuNPs or Au–IrO_2_ NFs

In order to obtain bacterial growth curves (Figures 2c and 4c), 40 μL of 20 nm AuNPs (2.5 nM) or Au–IrO_2_ NFs (0.24 nM) coated with BSA were mixed with 160 μL of *A. fischeri* (10^3^ cfu/mL) in the transparent 96-well microplate with a lid. Additionally, 40 μL of pesticide tributyltin (at a concentration of 100 ng/mL) was mixed with 160 μL of *A. fischeri* (about 10^3^ cfu/mL) as a positive-toxic control. The microplate was then placed on the support of SpectraMax iD3 and absorbance at 600 nm was detected every 10 min for 48 h under low speed of orbital shaking. And the bioluminescence signal of growing *A. fischeri* was collected at 490 nm every 10 min for 48 h by using white 96-well microplates with lids. All the measurements were performed using three replicates.

### Characterization of *A. fischeri* and Metallic Nanoparticles

To characterize the internal structure, external morphology, dispersion, diameter, and size uniformity of AuNPs and Au–IrO_2_ NFs, high-resolution transmission electron microscopy measurements were carried out using a Tecnai G2-F20 instrument (Figure S5a and 4b). TEM grids (carbon film 300 MESH Copper grids CF300-CU) were obtained from Electron Microscopy Sciences. For TEM imaging, metallic nanoparticles coated with BSA (AuNPs and Au–IrO_2_ NFs) were first centrifuged and then diluted with milli-Q water until a transparent color was achieved. The diameters of the nanoparticles and their size distribution were analyzed using ImageJ software and are represented as a histogram (Figures S5b and S8a). SEM images of *A. fischeri* were obtained using a SEM Zeiss EVO MA10. A 20 mL aliquot of 10^9^ cfu/mL fresh *A. fischeri* was processed according to SEM testing protocol,^55^ which enabled the clear visualization of the bacteria’s morphology and structure (Figure 2d). Cryogenic electron microscopy was used to visualize *A. fischeri* with metallic nanoparticles (AuNPs and Au–IrO_2_ NFs) using a TEM JEOL 2011 200 kV (Figures 2e and 4e). For the measurement, 10^9^ CFU/mL *A. fischeri* and metal nanoparticles coated with BSA (1.25 nM AuNPs and 0.06 nM Au–IrO_2_ NFs) were mixed in equal volumes and processed for Cryo-TEM testing. A 3.9 μL aliquot of the resuspended was added to a carbon TEM grid, held with a pair of forceps, and loaded onto a preparation chamber containing a liquid ethane bath cooled to a temperature between −178 and −180 °C using an automated liquid nitrogen flow.^56^ The resulting images were acquired by a Gatan Ultrascan US1000 CCD camera and analyzed with a Digital Micrograph 1.8.

### Conjugation of Metallic Nanoparticles with Antibodies

The metallic nanoparticles were conjugated with antihuman IgG antibodies (anti-HIgG) or antinucleoprotein of SARS-CoV-2 antibodies (anti-Np) by following a previously established protocol.^42,47^ Briefly, AuNPs solution was adjusted to pH 8 and Au–IrO_2_ NFs solution to pH 7 using 0.1 M borate buffer (BB, pH 9.2) for conjugation with anti-HIgG and anti-Np, respectively. And then 1.5 mL of AuNPs or Au–IrO_2_ NFs were mixed with 100 μL of 30 μg/mL of biotinylated anti-HIgG (or 100 μL of 10 μg/mL of anti-Np) and incubated for 30 min with 550 rpm shaking at room temperature. Next, 100 μL of 1% BSA (w/v) solution was added, and the mixture was incubated for another 30 min with 550 rpm shaking at RT. The AuNPs conjugates were centrifuged at 14 000 rpm (17 530 rcf) and RT for 20 min, while Au–IrO_2_ NFs conjugates were centrifuged at 6000 rpm (3220 rcf) and RT for 10 min. We removed the supernatants were discarded, and the pellets of nanoparticles conjugates were washed one time by using equal volume of PBST (0.01 M PBS, 0.05% Tween-20, pH 7.4). And then the pellets were centrifuged again using the same parameter and the pellets were resuspended in 0.75 mL (AuNPs conjugates) or 1.5 mL (Au–IrO_2_ NFs) of PBS (0.01 M, pH 7.4) and stored at 4 °C for further use. 1.25 nM of AuNPs and AuNPs conjugates aqueous solution (or 0.12 nM of Au–IrO_2_ NFs and Au–IrO_2_ NFs conjugates) were used for the DLS and particle surface charge measurement (*Z*-potential) (Figures S5c, S8b and Table S1). These measurements allowed us to estimate the changes of size, distribution, and stability of metallic nanoparticles before and after conjugation with protein. A PCMT ThermoShaker (Grant Instruments, UK) was used for all of the incubation steps. Metallic nanoparticles were centrifuged in a Centrifuge Allegra 64 R from Beckman Coulter (USA). DLS and *Z*-potential measurements were performed using ZetaSizer Nano ZS (Malvern, United Kingdom).

### Detection of HIgG and SARS-CoV-2 Nucleoprotein Based on Colorimetric ELISA

The colorimetric ELISA was operated by following previously published paper,^54^ Briefly, 100 μL of 2 μg/mL of anti-HIgG or 5 μg/mL of anti-Np in CBS (0.05 M, pH 9.6) was added to each well and incubated overnight at 4 °C for coating the capture antibodies on the wells. The solution with the antibody was removed, and the wells were washed three times with 250 μL of washing buffer PBST (0.01 M PBS, 0.05% Tween-20, pH 7.4). Next, 200 μL of 3% BSA (in 0.01 M PBS, pH 7.4) was added for blocking extra space and avoiding the nonspecific interactions. The plate was incubated for 1 h at 37 °C followed by washing steps. For the assay, 100 μL of HIgG or nucleoprotein samples were added to the wells precoated with anti-HIgG or anti-Np, and the plate was incubated for 45 min at 37 °C. Afterward, 100 μL of detection anti-HIgG or anti-Np antibody were added to each well and incubated for 30 min at 37 °C. Next, 100 μL of streptavidin-HRP or secondary antibody modified by HRP was added to each well and incubated for another 30 min at 37 °C. After each incubation step, the plate was washed three to five times with PBST. Finally, 100 μL of TMB (substrate solution) was added to each well and incubated for 20 min at 37 °C, followed by the addition of 50 μL of 1 M H_2_SO_4_ (stop solution). The plate was immediately put in a spectrophotometer, and the absorbance of solution in the wells was detected at 450 and 620 nm.

### Detection of HIgG or Nucleoprotein Based on BBLISA (AuNPs or Au–IrO_2_ NFs)

The first three steps of the BBLISA protocol, which involve capture antibodies precoating, blocking with BSA, and analyte incubation, are identical to those in the colorimetric ELISA method. However, in BBLISA, the analyte-bound wells are incubated with AuNPs (2.5 nM) or Au–IrO_2_ NFs (0.12 nM) conjugates for 30 min at 37 °C, followed by washing five times. Finally, 100 μL of precultured bioluminescent bacteria (*A. fischeri*, 10^9^ cfu/mL) were added to the wells, and bioluminescence is immediately collected using a spectrophotometer at 495 nm.

### Data Analysis

Colorimetric or bioluminescence signals were acquired with SpectraMax iD3. Fiji ImageJ-windows 64 bit was used to measure the diameter of nanoparticles from TEM images. Origin 2019-64 bit software was used for fitting curves using four parameter logistic equation.^42,46,47^

The relative bioluminescence signal change (%) in BBLISA (Figures 2, 3, 4 and 5, S3b and S8b) at a given absorber or target concentrations were calculated according to the following formula
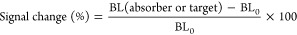
where BL (absorber or target) is the bioluminescence *A. fischeri* in the presence of the absorber or the target; BL_0_ is the bioluminescence of *A. fischeri* in the absence of absorber or target.

The calibration curves for BBLISA were fit with the following four parameter logistic equation
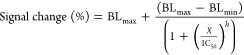
where *X* is the concentration of the target, BL_max_ is the maximum value of the signal change (%) in the absence of target, BL_min_ is the minimum value of the signal change (%) in the presence of the target, IC_50_ is the concentration of target where is located the inflection point, and h is the Hill coefficient which describes the slope of the curve.

To improve the comparison between BBLISA and ELISA, The relative bioluminescence signal changes (%) (Figures 3 and 5) were normalized by using the following equation for the performance comparison between BBLISA and ELISA.
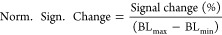


Additionally, the initial absorbance (Figures 3 and 5) and bioluminescence signals were normalized by using the following formulas
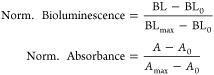
where BL, BL_0_ and BL_max_ represent the bioluminescence in the presence of the target, in the absence of target and at saturating concentration of target, respectively. *A*, *A*_0_ and *A*_max_ represent the absorbance in the presence of the target, in the absence of target, and at saturating concentration of target, respectively.

The LOD is calculated by following the equations
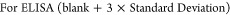

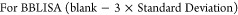


The different sign in the equations is due to the different sign of the signal change (%), which is a signal-on for ELISA and a signal-off for BBLISA. All of the parameters describing the analytical performance of the assays are summarized in Table 1.

## Supporting Information Available

bioluminescence signal changes of *A. fischeri* in the presence of different sizes of AuNPs; raw absorption spectra of different sizes of AuNPs; normalized growth curves of *A. fischeri* in presence or absence of AuNPs; bioluminescence signal changes of different concentrations of *A. fischeri* in the presence of different concentrations of AuNPs; characterization of AuNPs before or after conjugation with antibodies; calibration curves (raw bioluminescence signal) for detection of HIgG and SARS-CoV-2 nucleoprotein using AuNPs-based BBLISA (BBLISA_AuNPs) platform; calibration curves (raw absorbance) for detection of HIgG and SARS-CoV-2 nucleoprotein using a classic immunosandwich ELISA platform; characterization of Au–IrO_2_ NFs before or after conjugation with antibodies; normalized growth curves of *A. fischeri* in presence or absence of Au–IrO_2_ NFs; bioluminescence signal changes of different concentrations of *A. fischeri* in the presence of different concentrations of Au–IrO_2_ NFs; calibration curves (raw bioluminescence signal) for detection of HIgG and SARS-CoV-2 nucleoprotein using Au–IrO_2_ NFs-based BBLISA (BBLISA_Au–IrO_2_ NFs) platform; summary of characterization parameters (*Z*-Average, polydispersity index, and zeta potential) of AuNPs and Au–IrO_2_ NFs before and after conjugation with antibodies; comparison of the spike and recovery experiments for the target HIgG using BBLISA_AuNPs and BBLISA_Au–IrO_2_ NFs; comparison of the spike and recovery experiments for the target SARS-CoV-2 nucleoprotein using BBLISA_AuNPs and BBLISA_Au–IrO_2_ NFs; cost summary of AuNPs- and Au–IrO_2_ NFs-based BBLISAs for the detection of HIgG and SARS-CoV-2 nucleoprotein; comparison of Au–IrO_2_ NFs-based BBLISA and commercial ELISA and LFIA for the detection of HIgG and SARS-CoV-2 nucleoprotein (PDF)
