# Supplementary material for: Harnessing Bioluminescent Bacteria to Develop an Enzymatic-free Enzyme-linked immunosorbent assay for the Detection of Clinically Relevant Biomarkers
Source: ACS Appl Mater Interfaces. 2024 Apr 23;16(24):30636–47. doi: 10.1021/acsami.4c01744 (PMC11194763; doi:10.1021/acsami.4c01744)
Supplement: Supplementary file 1 — am4c01744_si_001.pdf [file am4c01744_si_001.pdf]

Supporting Information

Harnessing Bioluminescent Bacteria to Develop an Enzymatic-Free ELISA for the detection of Clinically Relevant Biomarkers

Liming Hu,[1] Marianna Rossetti,[1] José Francisco Bergua,[1] Claudio Parolo,[2] Ruslan Álvarez-Diduk,[1] Lourdes Rivas,[1] Andrea Idili,[3] Arben Merkoçi*[1,4]*

[1]Nanobioelectronics & Biosensors Group, Catalan Institute of Nanoscience and Nanotechnology (ICN2), CSIC and BIST, Campus UAB, Bellaterra, 08193, Barcelona, Spain

[2]Barcelona Institute for Global Health (ISGlobal), Hospital Clínic-Universitat de Barcelona, 08036, Barcelona, Spain

[3]Department of Chemical Sciences and Technologies, University of Rome Tor Vergata, Via della Ricerca Scientifica, 00133, Rome, Italy

[4]Institució Catalana de Recerca i Estudis Avançats (ICREA), Passeig Lluís Companys 23, 08010, Barcelona, Spain

Corresponding Authors: *Arben Merkoçi: arben.merkoci@icn2.cat; *Andrea Idili: andrea.idili@uniroma2.it

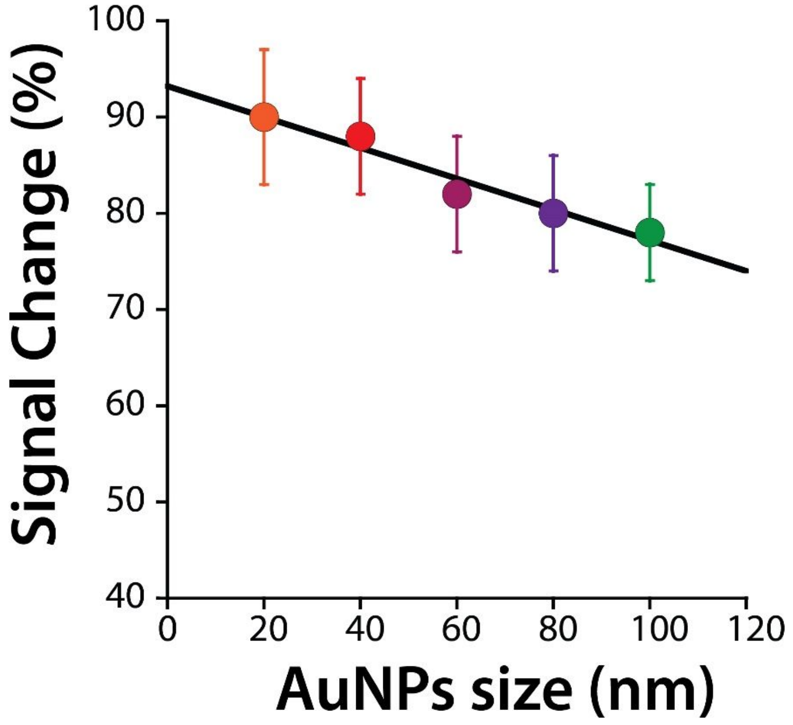

Figure S1. Bioluminescence signal changes of *A. fischeri* in presence of different sizes of AuNPs. AuNPs induce a suppression of the bioluminescence signal and the relative signal change (%) can be correlated with their size (20, 40, 60, 80 and 100 nm). This linear trend can be explained by the different overlap between the absorption spectra of AuNPs and the bioluminescence spectra of bacteria.

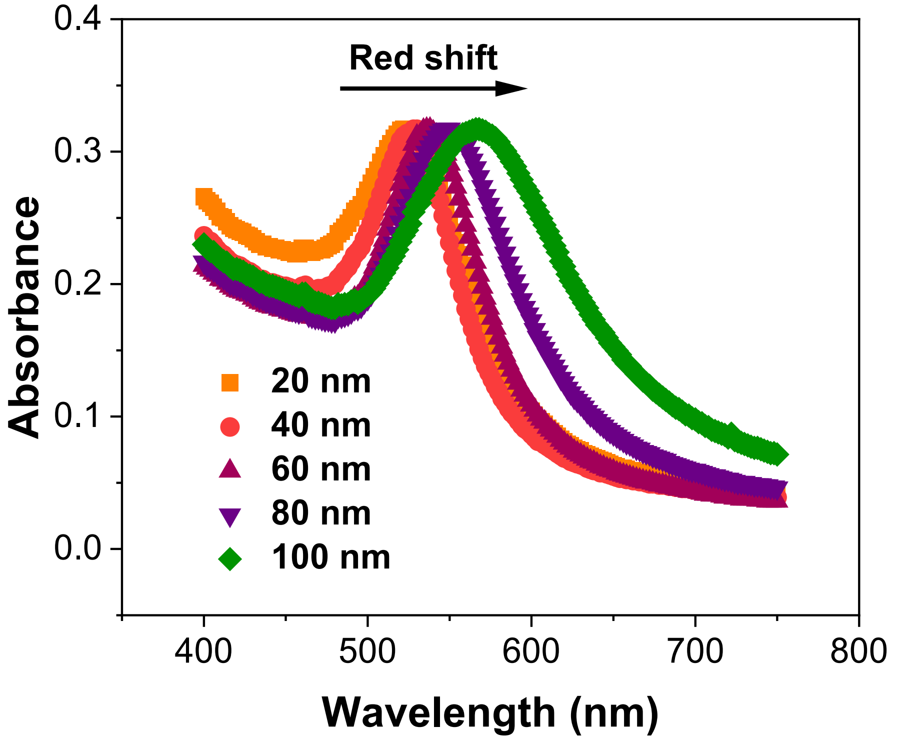

Figure S2. Raw absorption spectra of AuNPs of different sizes. To achieve the same identical absorbance values at their maximum absorption wavelengths we used different concentrations. Specifically, for AuNPs of 20 nm, 40 nm, 60 nm, 80 nm and 100 nm we used 1.30 nM, 0.11 nM, 0.03 nM, 0.015 nM and 0.007 nM, respectively.

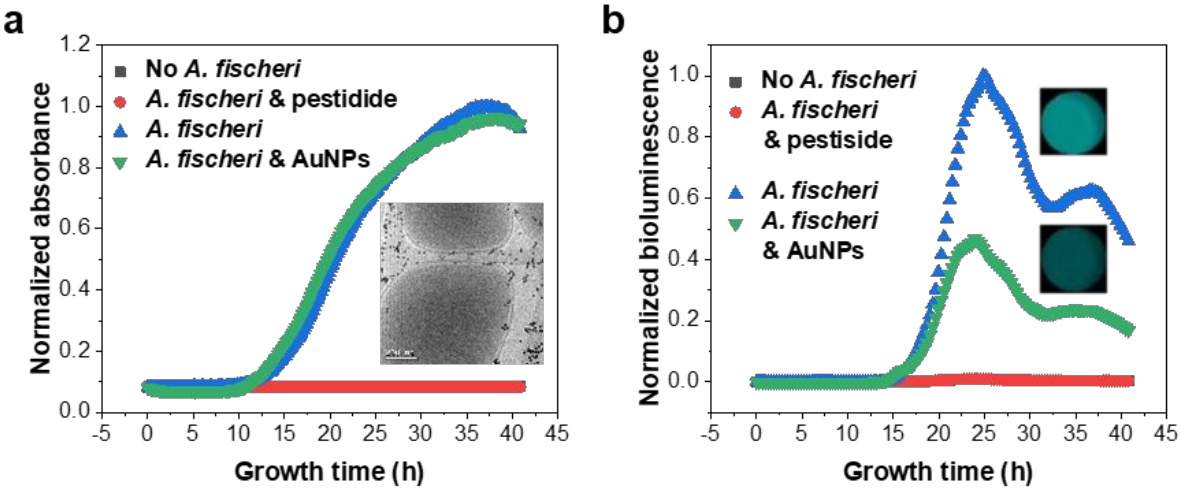

Figure S3. Normalized growth curves of *A. fischeri* in presence or absence of AuNPs. (a) The normalized absorbance at 600 nm of *A. fischeri* in the presence and absence of AuNPs (2.5 nM, green and blue curves) or the pesticide tributyltin (100 ng/mL, red curve) was measured from 0 h to 40 h. Note that the curves of No *A. fischeri* (black rectangle) is hidden by *A. fischeri* & pestiside (red circle) because they completely overlap and coincide with baseline. The inset shows a Cryo-TEM image showing that AuNPs are evenly distributed in the culture medium and some even adhere to the wall of *A. fischeri* without affecting their growth. (b) The normalized bioluminescence signal of *A. fischeri* in the presence and absence of 20 nm AuNPs was collected from 0 h to 40 h. Note that the curves of No *A. fischeri* (black rectangle) are obscured by *A. fischeri* & pestiside (red circle) because they are completely overlapped and coincide with the baseline. The embedded images were captured with a smartphone in a dark environment and show the bioluminescence ability of A. fischeri in the absence (top image, strong bioluminescence intensity) and presence (bottom image, weak bioluminescence intensity) of 20 nm AuNPs. The normalized bioluminescence values are

estimated as the ratio B/Bmax, where Bmax is the maximum bioluminescence signal during the growth period (0-40 h) and B is the bioluminescence signal at each detection time (growth time).

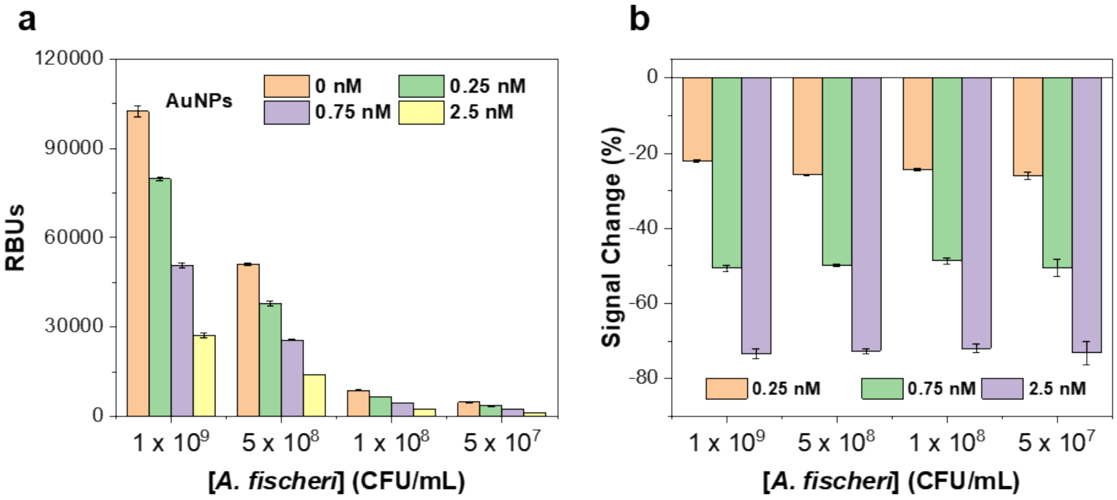

Figure S4. Bioluminescence signal changes of different concentrations of *A. fischeri* in the presence of different concentrations of AuNPs. (a) Raw bioluminescence signals collected from different solutions of *A. fischeri* (5 x 10^7, 1 x 10^8, 5 x 10^8 and 1 x 10^9 CFU/mL) in the absence and presence of 20 nm AuNPs (0.25, 0.75 and 2.5 nM). (b) The raw bioluminescence signals were then converted to signal change (%) (see Materials and Methods) for the different solutions of *A. fischeri* in the absence and presence of 20 nm AuNPs.

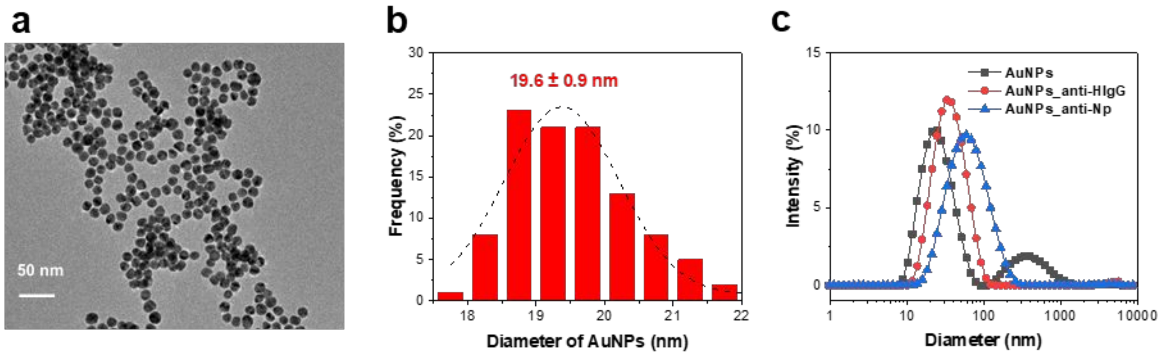

Figure S5. Characterization of AuNPs before and after antibody conjugation. (a) TEM image showing 20 nm AuNPs with spherical morphology and a homogeneous size and shape distribution. (b) Histogram plot of AuNPs with a size of 19.6 ± 0.9 nm (measured 100 particles from the TEM image using ImageJ software). The black dashed line represents the Gaussian fit used to plot the size distribution. (c) DLS (Dynamic Light Scattering) analysis of AuNPs before (black) and after conjugation with antibodies (red and blue).

Table S1. Z-Average, PDI (polydispersity index) and zeta potential of AuNPs and Au-IrO_2 NFs before and after conjugation with antibodies.

	**Z-Ave (nm)**	**PDI**	**Zeta Potential (mV)**
AuNPs	27.0 ± 0.5	0.351 ± 0.012	− 41.9 ± 0.3
AuNPs_anti-HIgG	31.6 ± 0.7	0.210 ± 0.007	− 35.4 ± 0.4
AuNPs_anti-Np	50.2 ± 0.6	0.267 ± 0.004	− 34.5 ± 0.5
Au-IrO_2 NFs	120.7 ± 1.6	0.122 ± 0.004	− 33.9 ± 0.3
Au-IrO_2 NFs_anti-HIgG	171.2 ± 2.7	0.159 ± 0.005	− 38.2 ± 0.3
Au-IrO_2 NFs_anti-Np	168.9 ± 5.7	0.163 ± 0.011	− 38.2 ± 0.6

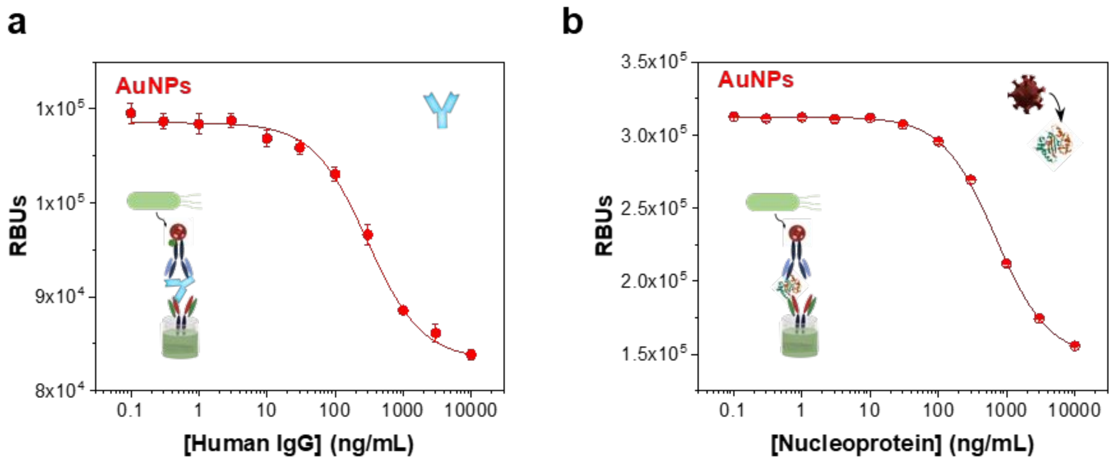

Figure S6. Calibration curves (raw bioluminescence signal) for detection of human IgG and SARS-CoV-2 nucleoprotein using AuNPs-based BBLISA (BBLISA_AuNPs) platform. (a) Calibration curve for the detection of human IgG (b) Calibration curve for the detection of SARS-CoV-2 nucleoprotein. Reported error bars reflect standard deviations derived from three independent wells.

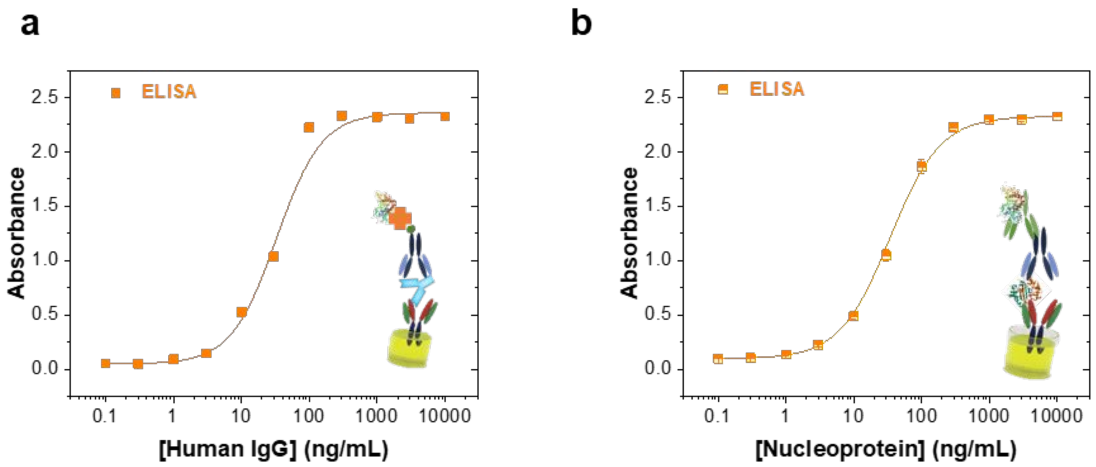

Figure S7. Calibration curves (raw absorbance) for detection of human IgG and SARS-CoV-2 nucleoprotein using a classic immunosandwich ELISA platform. (a) Calibration curves for the detection of human IgG (b) Calibration curves for the detection of SARS-CoV-2 nucleoprotein. Reported error bars reflect standard deviations derived from three independent wells.

Table S2. Comparison of the spike and recovery experiments for the target human IgG using BBLISA_AuNPs and BBLISA_Au-IrO_2 NFs. Estimated analyte concentrations are shown in Figures 3b and 5b.

Assay	Concentration of analyte (ng/mL)	Human IgG recovery (%)
BBLISA_AuNPs	50	85.8 ± 10.7
	100	112.8 ± 5.5
	200	85.8 ± 10.7
	300	92.9 ± 7.2
	500	83.4 ± 8.5
	1000	105.5 ± 3.2
	2000	86.5 ± 1.8
BBLISA_Au-IrO_2NFs	0.6	112.3 ± 9.7
	2	123.8 ± 14.1
	6	112.3 ± 8.6
	20	113.3 ± 8.1
	60	110.3 ± 5.2
	200	115.3 ± 2.0
	600	88.4 ± 7.0
	2000	97.0 ± 7.6

Assay	Concentration of analyte (ng/mL)	Nucleoprotein recovery (%)
BBLISA_AuNPs	20	92.5 ± 10.0
	30	111.2 ± 4.0
	60	84.0 ± 5.1
	100	104.1 ± 6.8
	200	102.7 ± 6.8
	300	100.7 ± 5.8
	600	103.9 ± 0.3
	1000	119.9 ± 5.0
BBLISA_Au-IrO_2NFs	3	91.9 ± 2.8
	6	94.1 ± 2.9
	30	82.3 ± 6.9
	60	83.3 ± 1.0

	200	81.0 ± 3.9
	300	96.1 ± 6.0
	600	104.9 ± 5.2

Table S3. Comparison of the spike and recovery experiments for the target SARS-CoV-2 nucleoprotein using BBLISA_AuNPs and BBLISA_Au-IrO_2 NFs. Estimated analyte concentrations are shown in Figures 3d and 5d.

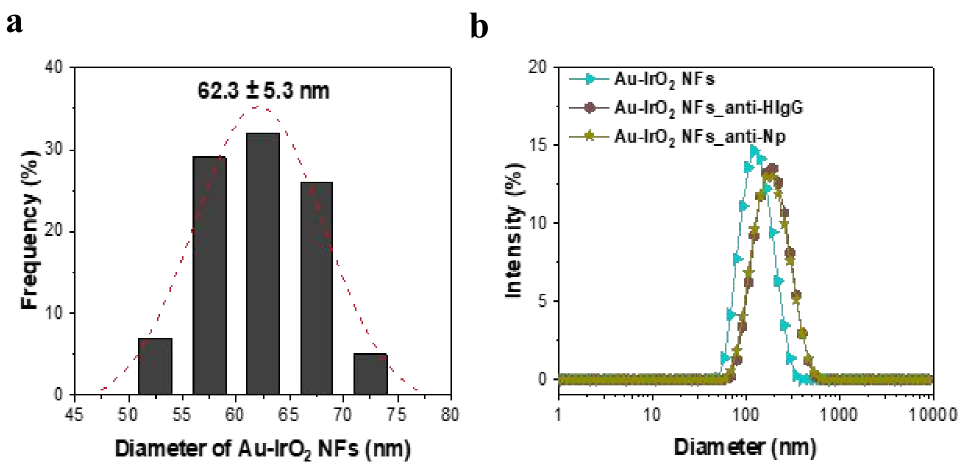

Figure S8. Characterization of Au-IrO$_2$ NFs before and after antibody conjugation. (a) Histogram graph of Au-IrO$_2$ NFs with a size of 62.3 ± 5.3 nm (measurements of 100 particles in the TEM image using ImageJ software). The red dashed line represents the Gaussian fit used to plot the size distribution. (b) Dynamic Light Scattering (DLS) analysis of Au-IrO$_2$ NFs before and after antibody conjugation.

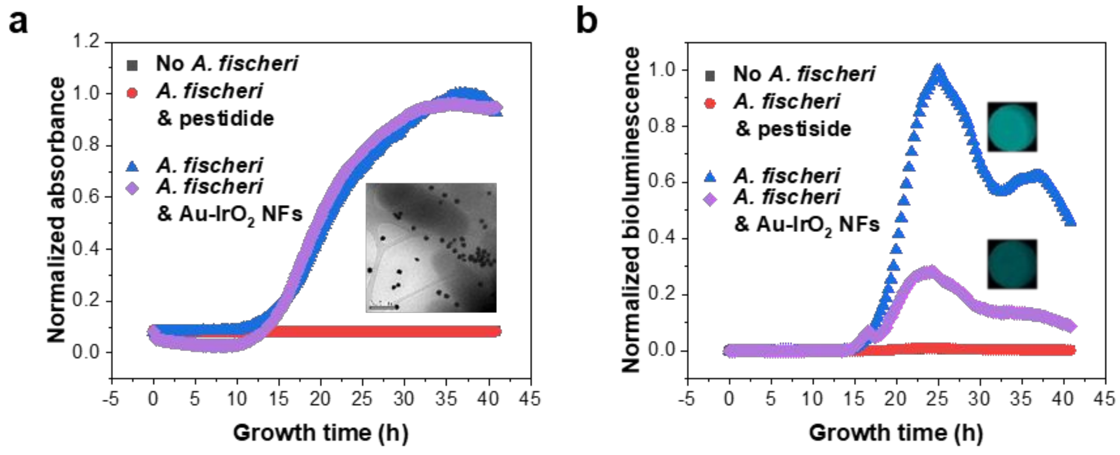

Figure S9. Normalized growth curves of *A. fischeri* in the presence or absence of Au-IrO_2 NFs. (a) The normalized absorbance at 600 nm of *A. fischeri* in the presence and absence of Au-IrO_2 NFs (0.24 nM, blue and purple curves) or the pesticide tributyltin (100 ng/mL, red curve) was measured from 0 h to 40 h. Note that the curves of no *A. fischeri* (black rectangle) is hidden by *A. fischeri* & pestiside (red circle) because they are completely overlapped and consistent with baseline. The inset shows a Cryo-TEM image, indicating that Au-IrO_2 NFs are evenly distributed in the culture medium and some even adhere to the wall of *A. fischeri* without affecting their growth. (b) Normalized bioluminescence signal of *A. fischeri* in the presence and absence of Au-IrO_2 NFs was collected from 0 h to 40 h. Of note, the curves of No *A. fischeri* (black rectangle) is hidden by *A. fischeri* & pestiside (red circle) because they are completely overlapped and consistent with baseline. The embedded images were captured by a smartphone in a dark environment and represent the bioluminescence capability of *A. fischeri* in the presence (bottom image, weak bioluminescence intensity) and absence (top image, strong bioluminescence intensity) of Au-IrO_2 NFs. The normalized bioluminescence values are estimated as the ratio B/B_{max}, where B_{max} represents the maximum bioluminescence signal during the growth period (0-40h), and B is the bioluminescence signal at each detection time (growth time).

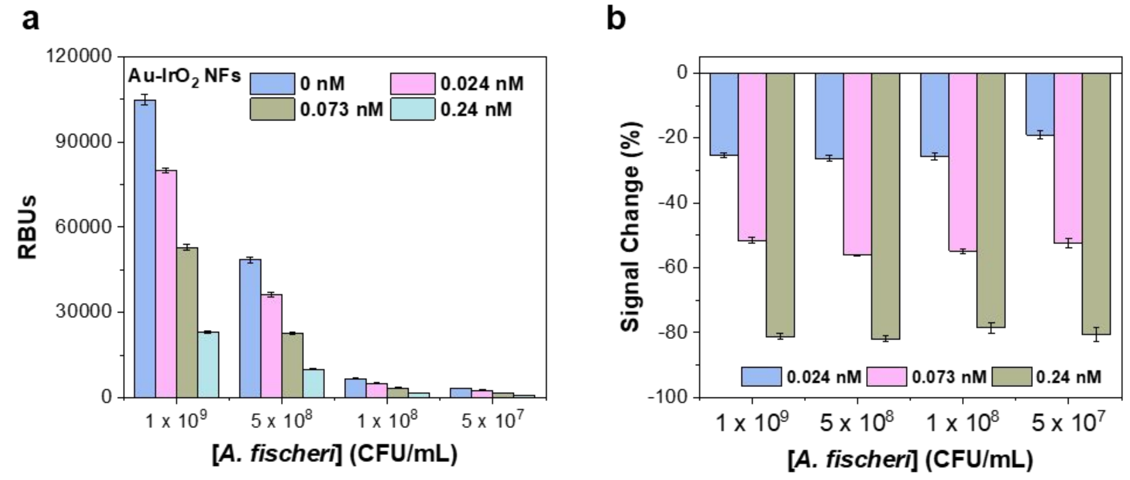

Figure S10. Bioluminescence signal changes of different concentrations of *A. fischeri* in the presence of different concentrations of Au-IrO_2 NFs. (a) Raw bioluminescence signals were collected from different solutions of *A. fischeri* ($5 \cdot 10^7$, $1 \cdot 10^8$, $5 \cdot 10^8$ and $1 \cdot 10^9$ CFU/mL) in the absence and presence of Au-IrO_2 NFs (0.024, 0.073 and 0.24 nM). (b) The raw bioluminescence signals were then converted to signal change (%) (see Data analysis ofExperimental methods) for the different concentrations of *A. fischeri* in the absence and presence of of Au-IrO_2 NFs.

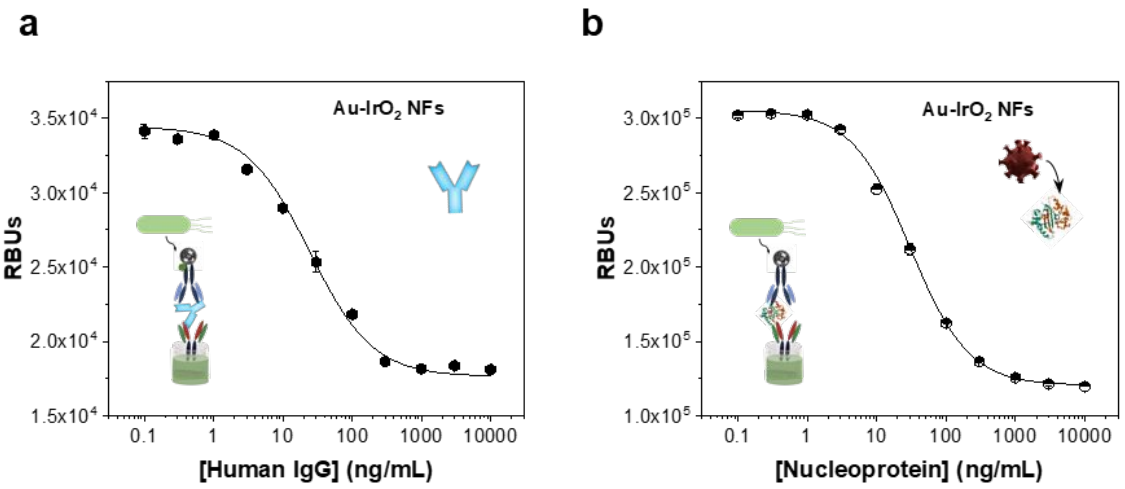

Figure S11. Calibration curves (raw bioluminescence signal) for the detection of human IgG and SARS-CoV-2 nucleoprotein using the Au-IrO_2 NFs-based BBLISA platform. (a) Calibration curve for the detection of human IgG. (b) Calibration curve for the detection of SARS-CoV-2 nucleoprotein. Error bars reported reflect standard deviations derived from three independent wells.

Table S4. Cost summary of AuNPs- and Au-IrO2 NFs-based BBLISAs for the detection of human IgG and SARS-CoV-2 nucleoprotein.

Materials name	Materials specification	Material price/unit	Amount/ 96 tests	Cost/96 tests (€)	Note
anti-HIgG, coating Abs (I1886)	3.0 mg/mL (2 mL)	173 €	19.2 μg	0.55	
anti-HIgG, detection Abs (B1140)	1 mg/mL (2 mL)	222 €	38.4 μg	4.26	AuNPs (HIgG)
anti-HIgG, detection Abs (B1140)	1 mg/mL (2 mL)	222 €	19.2 μg	2.13	Au-IrO_2 NFs (HIgG)
HIgG, target (I2511)	10 mg	122 €	4.35 μg	0.05	
Tetrachloroauric acid trihydrate (520918-1G)	1 g	208 €	3.78 mg	0.79	AuNPs
Tetrachloroauric acid trihydrate (520918-1G)	1 g	208 €	2.68 mg	0.56	Au-IrO_2 NFs
Iridium(III) chloride hydrate (203491-1G)	1 g	197 €	0.51 mg	0.10	
White 96-well plates (436110)	80 cases	318 €	1 case	3.97	
Typtone (T7293-1KG)	1000 g	498 €	0.048 g	0.02	
Yeast extract (Y0875-1KG)	1000 g	291 €	0.0288 g	0.01	
Glycerol (BioReagent, G2025-1L)	1000 mL	195 €	0.0288 mL	0.01	

Sodium chloride (S9888-1KG)	1000 g	47 €	0.192 g	0.01	
PBS tablets (P4417-100TAB)	100 tablets	188 €	1.25 tablets	2.35	
SARS-CoV-2 Np (40588-V08B)	1 mg	2,956 €	4.35 µg	12.86	
Anti-Np, mAbs (40143-MM08)	1 mg	2,924 €	12.8 µg	37.43	Au-IrO_2 NFs (HIgG)
Anti-Np, mAbs (40143-MM08)	1 mg	2,924 €	6.4 µg	18.71	Au-IrO_2 NFs (Np)
Anti-Np, pAbs (40588-T30)<br><br>*Aliivibrio fischeri* (700601 TM)	1 mg<br><br>1 vial	2,372 €<br><br>423 €	48 µg	113.86	Recycled indefinitely
				**12.02**	**AuNPs_HIgG**
				**171.17**	**AuNPs_Np**
				**9.76**	**Au-IrO_2 NFs_HIgG**
				**152.45**	**Au-IrO_2 NFs_Np**

Table S5. Comparison of Au-IrO_2 NFs-based BBLISA and commercial ELISA and LFIA for the detection of human IgG and SARS-CoV-2 nucleoprotein.

Items	BBLISA_Au-IrO_2 NFs		ELISA _Human IgG	ELISA _SARS-CoV-2 nucleoprotein	LFIA_human IgG	LFIA_SARS-CoV-2 nucleoprotein
	Human IgG	Nucleoprotein				
**Procedure time (min)**	60	60	90/200	285/210/210	20	15-30
**Price (€/96tests)**	10 €	152 €	614/883 €	650/670/497€	133 € (10 tests)	153 €(25 tests)
**Incubation steps**	2 steps	2 steps	3/4 steps	3/3/4 steps	1 step	1 step
**LOD (ng/mL)**	0.4	0.6	0.24/0.94	0.069/0.035/0.12	20	98.5% and 100% (specificity and sensitivity)
**Range (ng/mL)**	2-250	3-255	1.6-100 (both)	0.07-50/0.09-6/0.39-25	20-10,000	
**Refs.**	This work	This work	BMS2091 (Thermo Fisher)/ LS-F31875-1(LSBio)	EH490RB (Thermo Fisher)/KIT40588 (SinoBiological)/STJE0006698 (St John's Laboratory)	LF-016-10 (Cytodiagnostics)	SARS-CoV-2 Rapid Antigen Test 2.0 (Roche)
